# Efficacy and safety evaluation of thromboprophylaxis strategies for central venous catheter-related thrombosis in cancer patients: a bayesian network meta-analysis and bibliometric analysis

**DOI:** 10.3389/fphar.2026.1786836

**Published:** 2026-04-14

**Authors:** Mingyu Meng, Qimeng Xu, Shiran Qin, Xiaoyu Chen, Congzhi Qin, Muling Shen, Dandan Xu, Shiyao Pang, Jingsi Zhong, Yuan Fan, Yongxia Xu, Qin Yi, Henghai Su, Jian Qin

**Affiliations:** 1 Department of Pharmacy, Guangxi Academy of Medical Sciences and the People’s Hospital of Guangxi Zhuang Autonomous Region, Nanning, China; 2 Department of Pharmacy, Guangxi Medical University, Nanning, China; 3 Department of Pharmacy, Zhuzhou Central Hospital, Zhuzhou, China; 4 Department of Radiotherapy III, Clinical Oncology Center, Guangxi Academy of Medical Sciences and the People’s Hospital of Guangxi Zhuang Autonomous Region, Nanning, China

**Keywords:** anticoagulation, bibliometrics, cancer, central venous catheter, meta-analysis

## Abstract

**Background:**

Catheter-related thrombosis (CRT) is a common complication following central venous catheter (CVC) placement in patients with cancer, increasing mortality and adverse outcomes. However, robust evidence on optimal prophylactic anticoagulation strategies remains limited.

**Methods:**

A bibliometric analysis was performed on English-language publications on CVC-associated thrombosis prevention in cancer patients indexed in the Web of Science (2000–2025). Descriptive analyses were conducted using the Bibliometrix package in R, with visualizations generated by VOSviewer and CiteSpace. For the network meta-analysis, clinical studies on prophylactic anticoagulation for CRT in cancer patients were systematically searched (up to 28 May 2025), and a Bayesian network meta-analysis was performed using R packages netmeta and gemtc.

**Results:**

A total of 680 publications from 52 countries were identified. The United States led in both publication output and citations, and research focus shifted from warfarin and low-molecular-weight heparin (LMWH) toward direct oral anticoagulants (DOACs). Nineteen clinical studies were included in the network meta-analysis. Compared with no prophylaxis, apixaban reduced CRT incidence (OR 0.31, 95% CI 0.17–0.54), and vitamin K antagonists (VKA), rivaroxaban, and LMWH also showed significant reductions. VKA was associated with a higher bleeding risk than apixaban (OR 2.29, 95% CI 1.08–4.98) and no prophylaxis. No significant differences were found for major bleeding, all-cause mortality, or adverse events among other treatments.

**Conclusion:**

Apixaban, VKA, rivaroxaban, and LMWH effectively prevent CRT, while VKA is associated with an increased bleeding risk. These findings support the favorable effect of DOACs on CRT prevention. Long-term safety data from large-scale, multicenter trials are still needed.

**Systematic Review Registration:**

https://www.crd.york.ac.uk/prospero/, identifier CRD420251218825.

## Graphical Abstract

**Figure:**
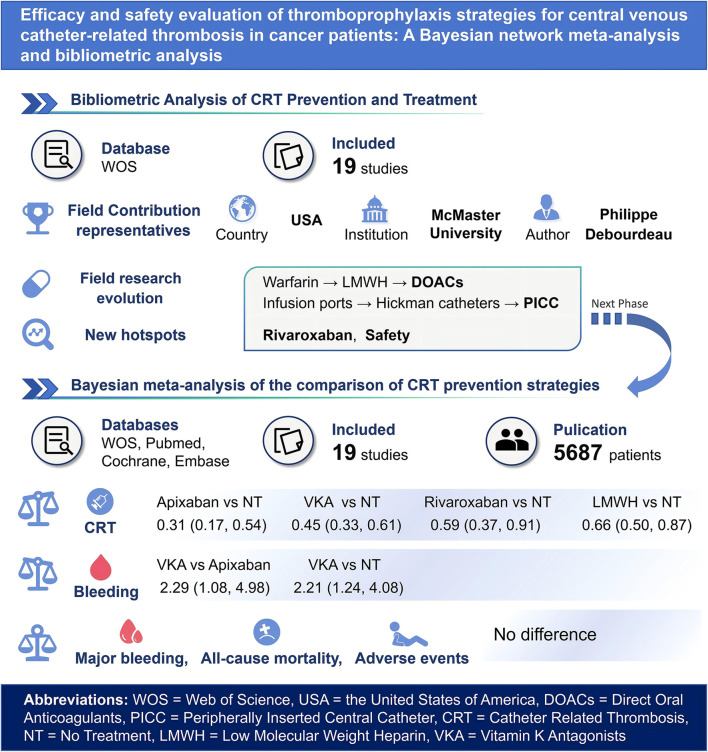

## 1 Introduction

With changes in human lifestyles and behavioral patterns, cancer has become one of the leading diseases threatening global public health. During chemotherapy, the strong irritant properties of antineoplastic agents may cause tissue and organ damage or result in drug extravasation. The use of central venous catheter (CVC) allows the safe administration of chemotherapeutic agents and effectively reduces the risk of local tissue injury and extravasation. To ensure the continuous delivery of chemotherapy, parenteral nutrition, or blood transfusion, patients with malignancies often require long-term CVC placement. Common types of CVC include peripherally inserted central catheter (PICC), tunneled central venous catheters, and totally implantable venous access ports (Ports). However, Catheter Related Thrombosis (CRT), as one of the common complications after central venous catheterization, has become an important factor affecting the continuity of treatment and clinical efficacy of cancer patients. Studies have shown that the average incidence of CVC-related thrombosis in cancer patients is about 5%–20%, and there is a certain mortality (CLOTS Trials Collaboration, 2014; Zhang et al., 2018), which is one of the risk factors leading to the death of cancer patients (Farge et al., 2016). Thrombotic events can increase the risk of disability, prolong hospitalization, interrupt intravenous chemotherapy, and exacerbate disease burden, thereby shortening patient survival and imposing substantial economic costs. CRT may also lead to catheter dysfunction, increased susceptibility to infection, and treatment interruption. In rare cases, it can result in post-thrombotic syndrome, cause further venous damage, or trigger life-threatening complications such as pulmonary embolism (PE), ultimately endangering patient survival (Geerts et al., 2004; Langer and Bokemeyer, 2012).

Prophylactic anticoagulation has been proposed as an effective strategy to reduce the incidence of CVC-related thrombosis by inhibiting the coagulation cascade and decreasing thrombus formation around the catheter (Khorana, 2013; Mosarla et al., 2019). However, current guidelines still have different recommendations on preventive anticoagulation for CVC-related thrombosis in cancer patients. The Chinese Guidelines for the Prevention and Treatment of Thrombotic Diseases (Chinese Thrombosis Guidelines Expert Committee, 2018) recommend venous thromboembolism risk assessment for patients with central venous catheter. For patients with moderate or high risk and without anticoagulation contraindications, it is recommended to use low-molecular-weight heparin (LMWH) or edoxaban for CRT prevention. The American Society of Clinical Oncology (ASCO) (Lyman et al., 2015) advises prophylactic-dose LMWH, unfractionated heparin, or rivaroxaban for patients with a Khorana score ≥3 or those with pancreatic, lung, or gastric cancer. In contrast, the European Society for Medical Oncology (ESMO) (Falanga et al., 2023) and the American Society of Hematology (ASH) (Lyman et al., 2021) do not recommend the use of drugs to prevent CRT. The above situation indicates that there is still a lack of sufficient research evidence to support the clinical application of prophylactic anticoagulants and reach a consensus in guidelines. Therefore, more evidence is needed to support whether CRT prevention is necessary and which prevention strategy is safer and more effective (to maintain the balance between the efficacy of thrombosis prevention and the risk of bleeding, and to increase the benefit of patients).

In this context, systematically reviewing the research progress and evidence gaps in this field is of great value. Bibliometric analysis can systematically map the research landscape, evolutionary trends and research hotspots in the field of prophylaxis for central venous catheter-related thrombosis in cancer patients; it not only clarifies the characteristic shift of mainstream anticoagulant strategies from traditional agents (warfarin, LMWH) to direct oral anticoagulants (DOACs) in this field, but also identifies the current evidence gaps and controversial issues lacking comprehensive quantitative comparison of multiple interventions. This provides a clear theoretical basis and research direction for the subsequent conduct of network meta-analysis, rendering the selection of interventions, setting of outcome indicators and determination of research priorities in the network meta-analysis more targeted and evidence-based. Network meta-analysis, on the other hand, can integrate both direct and indirect comparison evidence, quantitatively evaluating the differences in efficacy and safety among various prophylactic anticoagulation strategies. Therefore, this study intends to use bibliometric methods to analyze the landscape and development trajectory of CVC-related thrombosis anticoagulation in cancer patients, and to apply Bayesian network meta-analysis to comprehensively evaluate the efficacy and safety of different anticoagulants in preventing CRT. The aim is to provide evidence-based support for prophylactic anticoagulation decision-making, improve cancer patient prognosis, and offer future research directions in the field of CVC-related thrombosis anticoagulation.

## 2 Methods

The current study follows the PRISMA statement (Page et al., 2021) (Supplementary Table S1), The complete study protocol was pre-registered at PROSPERO with the registration number CRD420251218825. As no personal patient information is involved, ethical approval is not required for this study.

### 2.1 Search strategy

The Web of Science Core Collection database was searched, with the citation index set to Science Citation Index Expanded (SCI-Expanded), to construct the search strategy for CVC-related thrombosis prevention and treatment in cancer patients. All relevant literature from 2000 to 16 April 2025, was retrieved, limited to “Article” and “Review article” types, and restricted to English language. The search strategy is detailed in Supplementary Table S2.

Search the PubMed, The Cochrane Library, Embase and Web of Science databases. The search time range extended from database inception to 28 May 2025. The search focused on clinical studies regarding prophylactic anticoagulation for CVC-related thrombosis in cancer patients, with no language restrictions. The search included keywords such as “anticoagulation,” “central venous catheter”, and “cancer.” Additional keywords were derived by reviewing relevant literature appendices, and a combined approach of subject terms and free terms was used to construct the search strategy. The detailed search strategy is provided in Supplementary Table S3.

The bibliometric analysis was conducted using the Web of Science (WoS) Core Collection, a widely regarded and standardized source for bibliometric data. WoS provides consistent metadata, such as citation counts, author affiliations, and indexed keywords, essential for constructing co-authorship networks, co-citation maps, and keyword co-occurrence analyses. These features make WoS an ideal choice for bibliometric mapping. Owing to the inherent functional limitations of bibliometric analysis software, which only support single-database operations, the bibliometric component of this study exclusively included the WoS Core Collection.

### 2.2 Eligibility criteria

The inclusion criteria for this study were as follows: (1) study population: Patients with malignancies who have undergone CVC (Central Venous Catheter) placement, aged ≥18 years, and received prophylactic anticoagulation during CVC placement. The tumor types include both hematologic and solid tumors; (2) interventions: All prophylactic anticoagulants were included in the search. The DOACs mainly included rivaroxaban, apixaban, edoxaban, and dabigatran; low molecular weight heparins (LMWH) included enoxaparin, dalteparin, nadroparin, etc.; vitamin K antagonists (VKAs) included warfarin and other coumarin and indanedione derivatives; (3) outcomes: The primary outcomes were venous thromboembolism (VTE) and major bleeding events. The secondary outcomes included all bleeding events, all-cause mortality events, and adverse events. VTE was defined as a composite of pulmonary embolism (PE) and deep venous thrombosis (DVT), including both symptomatic and asymptomatic cases. The occurrence of CRT must be confirmed through diagnostic methods. PE diagnosis included computed tomography (CT), D-dimer, CT pulmonary angiography, and ventilation/perfusion scanning. Diagnosis of symptomatic or asymptomatic DVT included color Doppler ultrasound, venography, D-dimer, CT venography, magnetic resonance venography, radioactive iodine fibrinogen uptake test, and impedance plethysmography. Major bleeding events, bleeding events, and adverse events were defined according to the standards of each study (bleeding events encompass all bleeding conditions, including major bleeding; adverse events included any non-bleeding adverse events). Death was defined as all-cause mortality, including both catheter-related thrombosis-related death and death due to cancer progression; (4) study types: randomized controlled trials (RCTs) or non-randomized controlled trials (Non-RCTs). Non-RCTs primarily included non-randomized concurrent controlled trials, self-controlled studies, historical controlled studies, etc.

Exclusion criteria were: (1) inconsistent research content, such as therapeutic anticoagulation; (2) duplicate studies; (3) single-arm studies; (4) inappropriate study types, such as case reports and cross-sectional studies; (5) studies with incomplete data or serious missing data.

### 2.3 Study selection and data extraction

Two researchers independently screened the literature, selected studies, extracted data, and cross-verified the information according to the inclusion criteria. Duplicate studies were first excluded using EndNote X9.1, followed by screening based on titles and abstracts, and full-text articles were reviewed to determine study inclusion. If there were disagreements between the two researchers, a third researcher would be involved to resolve the discrepancies. The data to be extracted from the included studies included: study information, study design, baseline characteristics, interventions, dosage and frequency, duration of intervention, follow-up time, and outcome measures.

### 2.4 Risk of bias assessment

The risk of bias for the included studies was assessed according to the type of study. Randomized controlled trials (RCTs): The risk of bias for RCTs was assessed using the Cochrane Risk of Bias tool, with seven evaluation items, including: (1) random sequence generation; (2) allocation concealment; (3) blinding of participants and personnel; (4) blinding of outcome assessment; (5) incomplete outcome data; (6) selective reporting; (7) other bias. Non-randomized studies (Non-RCTs): The risk of bias for non-randomized studies was assessed using the ROBINS-E (Risk of Bias In Non-randomized Studies of Exposures) (2022) tool, with seven evaluation items, including: (1) bias due to confounding; (2) bias in selection of participants into the study; (3) bias in classification of interventions; (4) bias due to deviations from intended interventions; (5) bias due to missing data; (6) bias in measurement of outcomes; (7) bias in the selection of the reported result. Evaluation process: Two researchers independently assessed each study according to the evaluation criteria. For each item, the risk of bias was categorized as “Low,” “High,” or “Unclear.” Any disagreements were resolved through discussion, and a risk of bias plots was generated using R 4.3.2.

### 2.5 Statistical analysis

This study used the Bibliometrix package in R 4.3.2 to perform statistical analysis of relevant information, including annual publication volume, countries, institutions, authors, journals, and citation counts (Aria and Cuccurullo, 2017). Annual publication trends, geographic distribution maps, and thematic trend graphs were plotted. Visualization analysis was conducted using VOSviewer software, and co-occurrence networks and collaboration timeline graphs were created (van-Eck and Waltman, 2010). CiteSpace software was used to generate journal dual-network plots (Synnestvedt et al., 2005).

Bayesian network meta-analysis was exclusively performed using the gemtc package in R, a commonly used tool for network meta-analysis under the Bayesian framework. For binary variables, odds ratios (ORs) with their 95% credible intervals (CrIs) were selected as the effect sizes. The consistency of results was assessed using P-values, where P > 0.05 indicated good consistency, and a consistency model was chosen; if P < 0.05, indicating inconsistency between nodes, an inconsistency model was used. A hierarchical Bayesian model was fitted using the Markov Chain Monte Carlo (MCMC) method, with 50,000 iterations, the first 20,000 used for annealing to eliminate the influence of initial values, and the remaining for sampling. Four chains were used for each model. The prior distributions in the Bayesian method and flexible modeling were applied to determine the optimal effect model. Convergence and model stability were assessed using trace density plots, and the Potential Scale Reduction Factor (PSRF). In the case of closed loops, transitivity assumptions were tested to check the consistency between direct and indirect comparisons, with systematic analysis conducted using node splitting analysis.

To intuitively present the cumulative ranking probability of each intervention across different outcome indicators, the netmeta package in R was used for auxiliary visualization: raw data were imported into this package, organized via the pairwise function to complete frequentist NMA analysis, and then Surface Under the Cumulative Ranking Curve (SUCRA) values were calculated and ranking plots were generated. This package was solely used for generating visual graphs, and the conclusions of this study were mainly derived based on the Bayesian analysis results from the gemtc package. The probability ranking of each intervention was calculated to determine the probability of each intervention being ranked first. The cumulative ranking probability area under the curve was calculated and reported. A SUCRA value of 1 indicates the optimal intervention, while a SUCRA value of 0 indicates the worst. A ranking plot was created to display the SUCRA values for all outcomes. When more than 10 clinical trials were included, publication bias was assessed using Stata 14 to adjust the funnel plot and identify small-sample effects.

## 3 Results

### 3.1 Search results

In this study, 680 articles related to CVC-related thrombosis prevention and treatment in cancer patients were retrieved and screened from the Web of Science, with an overview of the bibliometric process shown in Supplementary Figure S1. After summarizing the search results from all databases, a total of 1,626 articles were obtained for the network meta-analysis. All articles were imported into EndNote X9.1 for screening, and after removing duplicates, 1,340 articles remained. After the initial screening, irrelevant studies and those with inappropriate study types were excluded, leaving 123 articles. Finally, after full-text review, 19 eligible studies were included (Abdelkefi et al., 2004; Bern et al., 1990; Boraks et al., 1998; Brandt et al., 2022; Carrier et al., 2019; Cortelezzia et al., 2003; Couban et al., 2005; De-Cicco et al., 2009; Heaton et al., 2002; Ikesaka et al., 2021; Karthaus et al., 2006; Khorana et al., 2019; Lavau-Denes et al., 2013; Lv et al., 2019; Mismetti et al., 2003; Monreal et al., 1996; Niers et al., 2007; Verso et al., 2005; Young et al., 2009). The study flow diagram is shown in Supplementary Figure S2.

### 3.2 Study characteristics

A total of 19 clinical studies were included in the network meta-analysis, of which 16 were RCTs and 3 were Non-RCTs. A total of 5,687 cancer patients who underwent CVC placement were included. The control group was the no prophylaxis (No treatment) group (18 studies, n = 2,776). 5 prophylactic CVC-related thrombosis regimens were ultimately included: (1) rivaroxaban (3 studies, n = 414); (2) apixaban (2 studies, n = 610); (3) LMWH (9 studies, n = 1,159); (4) UFH (2 studies, n = 224); (5) VKA (8 studies, n = 1,011). The characteristics of the included studies are shown in Supplementary Table S4.

### 3.3 Quality assessment of included studies

The quality of the 19 included studies was assessed using the Cochrane Risk of Bias tool and the ROBINS-E (2022 version) tool. In the assessment results, the overall risk of bias for 2 RCTs was rated as “Unclear”. One study (Couban et al., 2005) was assigned a High risk of other bias due to a 60% interruption rate of study drug administration, which was mainly caused by clinical conditions during chemotherapy, yet the interruption rate was balanced across groups and had no impact on the primary study outcomes. Another study (Niers et al., 2007) was rated as High for other bias owing to a 25% missed diagnosis rate of the primary outcome (CRT), while the missed diagnosis was consistent across all groups and did not exert a significant effect on the final study results. The remaining studies were rated as “Low” or “Unclear” for each item based on whether they reported the relevant information. Overall, the included studies achieved an acceptable quality level; the identified high-risk bias items were balanced across groups, and the original studies confirmed that the aforementioned biases had no substantive impact on the study results. The quality assessments for RCTs and Non-RCTs are shown in Supplementary Figures S3, S4.

### 3.4 Bibliometric analysis results

#### 3.4.1 Global trends in annual publications and citations

**FIGURE 1:**
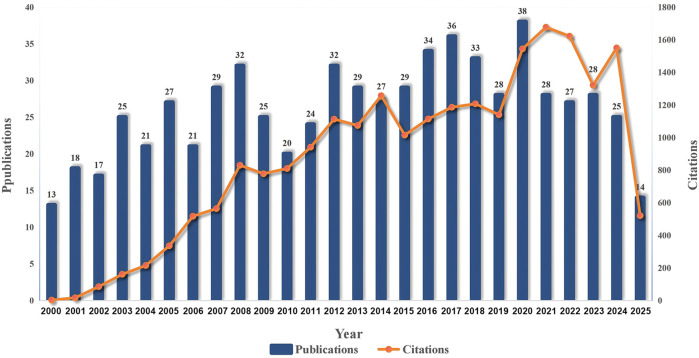
Annual publications of relevant literature.

**FIGURE 2:**
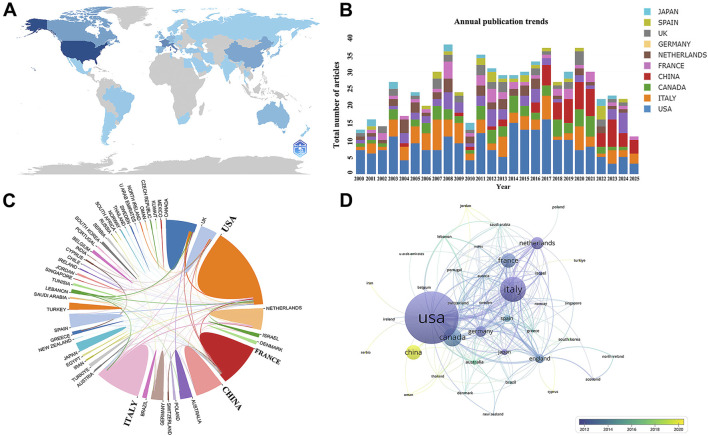
Country/region analysis. **(A)** Geographic Distribution Map of Total Publications by Country/Region; **(B)** Trend of Annual Publication Volume of the Top 10 Countries/Regions from 2000 to 2024; **(C)** Visualization of Countries/Regions Involved in International Collaboration; **(D)** Timeline of Country/Region Collaborations. The thickness of the lines reflects the intensity of collaboration, with color mapping based on the average year of collaboration. The color range from deep blue to yellow indicates the progression from the earliest to the most recent collaborations.

The trend of publications in the field of CVC-related thrombosis prevention and treatment in cancer patients is shown in Figures 1, 2. The annual publication volume exhibited a fluctuating upward trend, with small peaks in 2008, 2012, and 2017. A peak in publications occurred in 2020, followed by a slight decline in the next 5 years. The number of citations fluctuated in line with the publication trend, indicating continued attention from the medical community to this research area.

#### 3.4.2 Country/Region analysis

The 680 publications in the field of CVC-related thrombosis prevention and treatment in cancer patients involved 52 countries worldwide. An analysis of the top 10 countries by publication volume (Supplementary Table S5) showed that the United States ranked first in both publication volume (189 publications) and citation count (10,302 citations). Italy and China ranked second and third, respectively (Figures 2A,B). The MCP ratio indicated that the United Kingdom was more likely to collaborate with other countries. The visualization analysis results are shown in Figure 2. Publications on CVC-related thrombosis in cancer patients were mainly concentrated in North America, Asia, and Europe. The visual maps in Figures 2C,D illustrate that the United States has the most collaborations with other countries, especially with Italy, the United Kingdom, and others, while China is an emerging partner in collaboration.

#### 3.4.3 Institution and author analysis

A total of 1,153 institutions and 3,507 authors worldwide have participated in research on CVC-related thrombosis prevention and treatment in cancer patients, including many co-authored studies. Supplementary Tables S6, S7 provide statistics on the top 10 institutions and authors with the highest number of publications worldwide. The institution with the highest publication volume globally was McMaster University in Canada (36 publications), while the author with the highest publication volume was Philippe Debourdeau from France (16 publications; H-index = 10). The most cited author was Giancarlo Agnelli (1,170 citations). Co-occurrence analysis of institution and author collaborations (Supplementary Figure S5) revealed that McMaster University collaborates most frequently with other institutions. Philippe Debourdeau is the author with the most collaborations, forming the largest collaboration network centered around him. Considering publications, citations, and collaboration frequency, McMaster University emerges as the most authoritative and representative institution in this field, with Philippe Debourdeau being the most influential author.

#### 3.4.4 Influential journals and hot articles

**FIGURE 3:**
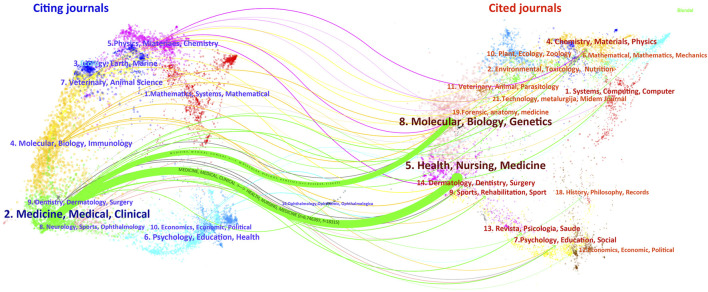
Journal dual overlay analysis.

The 680 publications were published in 274 journals, primarily focused on hematology and oncology journals. Supplementary Table S8 presents the top 10 journals by publication volume. The journals with the highest publication and citation counts were Thrombosis Research (30 publications) and Cochrane Database of Systematic Reviews (2,536 citations). Among the top 10 journals, five belong to the Q1 category in the Journal Citation Reports (JCR) ranking, with the most influential journal being Annals of Oncology (IF = 65.4). We found that the citation count of journals is more closely related to both the journal’s impact factor and its publication volume. Overall, Cochrane Database of Systematic Reviews is the most prominent journal in this field. To visualize the citation and co-citation relationships of journals and display the subject distribution of journals, a dual-map overlay analysis of journals was performed using CiteSpace. As shown in Figure 3, the two green-marked paths represent the primary citations, indicating that research published in molecular biology, genetics, and health-related journals is frequently cited by research in medicine, healthcare, and clinical studies, demonstrating the converging thematic development model.

An analysis of hot articles (Supplementary Table S9) revealed that the most cited article was a clinical study published by Antonio Palumbo from Italy in Leukemia in 2017 (Palumbo et al., 2008), which has been cited 651 times, averaging 36 citations per year. High-citation articles in this field primarily focus on the thrombotic risks associated with CVC in cancer patients.

#### 3.4.5 Research hotspot analysis

**FIGURE 4:**
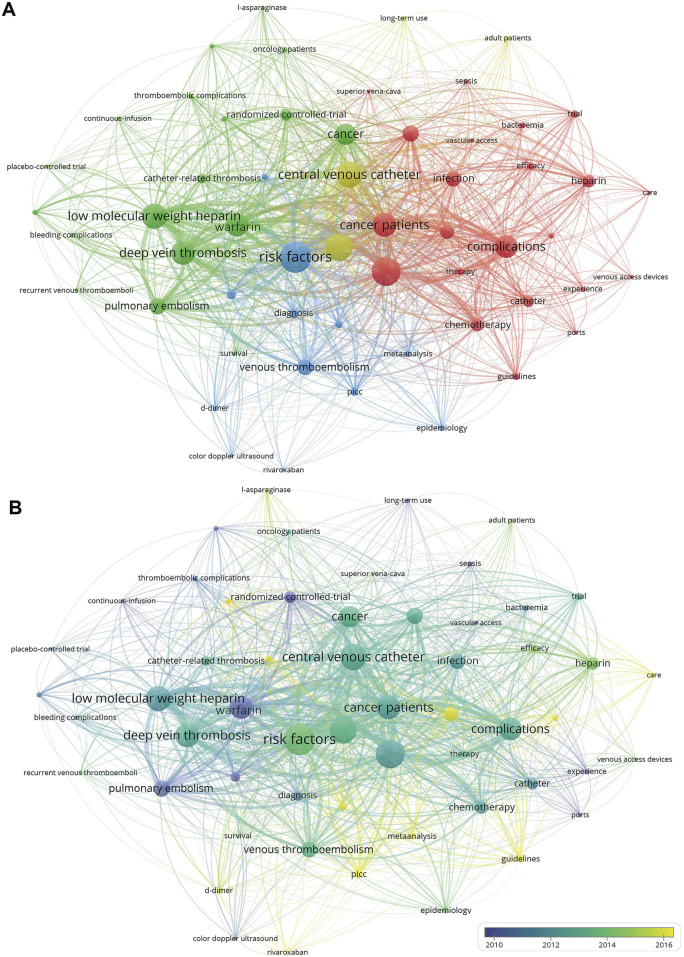
Keywords co-occurrence map. **(A)** Keyword Co-occurrence Map; **(B)** Keyword Timeline Chart.

Through keyword co-occurrence analysis, we can understand the areas of interest and emerging trends in the field of CVC-related thrombosis prevention and treatment in cancer patients. The keyword co-occurrence analysis (Figure 4) shows that the node size varies with the frequency of keyword appearances, and different colors are used to distinguish different clusters, with the distance between nodes representing the strength of association. As shown in Figure 4A, the results revealed that the keywords were divided into four clusters: the red and yellow clusters mainly focused on the management of CVC-related complications, such as “Complications,” “Management,” and “Thromboprophylaxis;” the green cluster centered on anticoagulation and prevention of catheter-related venous thrombosis, including keywords like “Low Molecular Weight Heparin” and “Warfarin;” the blue cluster focused on risk factors and diagnosis of thrombosis after catheter insertion, including “Risk factors” and “Diagnosis.” Figure 4 uses color mapping based on the average publication year of the keywords, from deep blue to yellow, representing research hotspots from earlier to more recent topics. Early hotspots primarily focused on “Warfarin,” “Pulmonary Embolism,” and “Ports,” while recent hotspots are mainly centered around “Rivaroxaban,” “PICC,” and “Meta-Analysis.”

By identifying high-frequency emerging keywords over a short period, we can precisely capture the frontier directions and dynamic changes in the research topics of anticoagulation for CVC-related thrombosis in cancer patients. Meanwhile, to address the limitations of the timeline visualization in Figure 4B; Supplementary Figure S6; Table 1 provide a more detailed analysis of the evolutionary trends in keyword research focus. Table 1; Supplementary Figure S6 showed that the earliest research topics were focused on “Ports” and “Hickman,” which were more widely studied CVC types requiring surgical implantation. “Risk-factors” appeared most frequently, indicating that catheter-related risk factors are a major area of focus. Regarding anticoagulants, “LMWH” was the longest-standing research hotspot, while “Warfarin” also experienced a prolonged research period. However, after 2016, research interest in Warfarin sharply declined, and a new hotspot, “Rivaroxaban”, emerged from 2020. “Safety” has also become a recent research hotspot, as the safety issues surrounding new drugs have become a significant research trend in the field.

**TABLE 1:** Keyword burst information.

Rank	Frequency of occurrence		Earliest year		Most Recent Year		Keywords with the longest research period
1	166	Risk-factors	2001	Ports	2020	Rivaroxaban	LMWH
2	154	Prevention	2001	System	2020	Safety	System
3	146	LMWH	2001	LMWH	2019	Definition	Cancer-patients
4	139	Deep-vein thrombosis	2001	Hickman	2017	Care	Chemotherapy
5	136	Complications	2001	Randomized trial	2016	Normal saline	Thrombosis
6	131	Cancer-patients	2001	Subclavian vein-thrombosis	2015	Clinical-practice guidelines	Complications
7	125	Central venous catheters	2002	Carcinoma	2015	Meta-analysis	Children
8	84	Pulmonary-embolism	2003	Experience	2014	PICC	Catheter
9	84	Thromboembolism	2004	Upper-extremity	2014	Epidemiology	Warfarin
10	72	Access devices	2004	Breast-cancer	2014	Guidelines	Prophylaxis

The above information only includes the top ten ranked keywords. The year represents the time when the keywords appeared.

### 3.5 Bayesian network meta-analysis results

#### 3.5.1 Network plots

**FIGURE 5:**
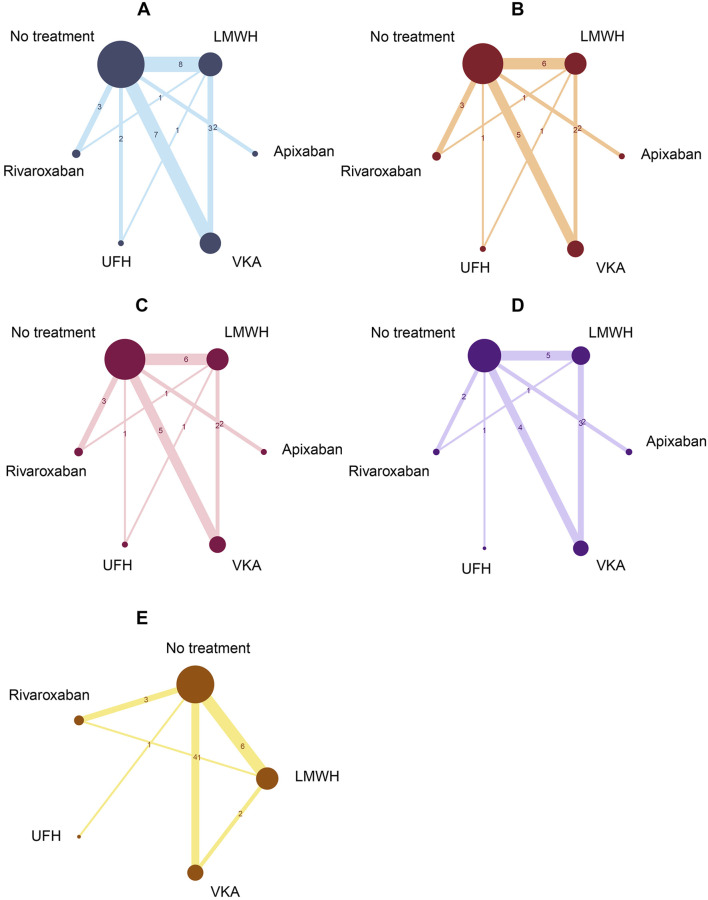
Network plots. Crl: credible interval; DOACs: direct oral anticoagulant; LMWH: low molecular weight heparin; OR: odds ratio; UFH: unfractionated heparin; VKA: Vitamin K Antagonist. **(A)** CRT. **(B)** Major bleeding. **(C)** Bleeding. **(D)** All-cause mortality. **(E)** Adverse events.

A total of 19 studies reported the outcome of CRT in cancer patients receiving prophylactic anticoagulation. 17 studies reported major bleeding outcomes. 17 studies reported bleeding outcomes. 12 studies reported all-cause mortality outcomes. And 14 studies reported adverse events. A network relationship diagram was formed for all five outcome indicators, centered around the “No treatment” group (Figure 5), which resulted in three closed loops.

#### 3.5.2 Selection and validation of the model

Due to the presence of closed loops in the network plots, nodes-splitting analysis was performed for consistency testing (Supplementary Figures S7, S8). For the CRT outcome, an inconsistency model was used for the UFH vs. LMWH comparison due to one inconsistency. For the other outcome indicators, all nodes showed P > 0.05, indicating good consistency, and consistency models were selected for major bleeding, bleeding, all-cause mortality, and adverse reactions for the network meta-analysis. Based on the chosen model types, 20,000 pre-iterations were set, followed by 50,000 iterations for analysis. Convergence tests were performed on the models. The trace density plots (Supplementary Figures S9–S13) and diagnostic plots (Supplementary Figures S4–S18) indicated that the MCMC chain trajectories for each outcome were stable, with the chains merging and overlapping. The density curves exhibited a normal distribution, the bandwidth values approached 0, Although the PSRF values for some outcome comparisons were marginally above 1.1 at the early stage of iterations, all parameters achieved adequate convergence with increasing iterations, confirming the stability and reliability of the model results.

#### 3.5.3 Network meta-analysis results

**FIGURE 6:**
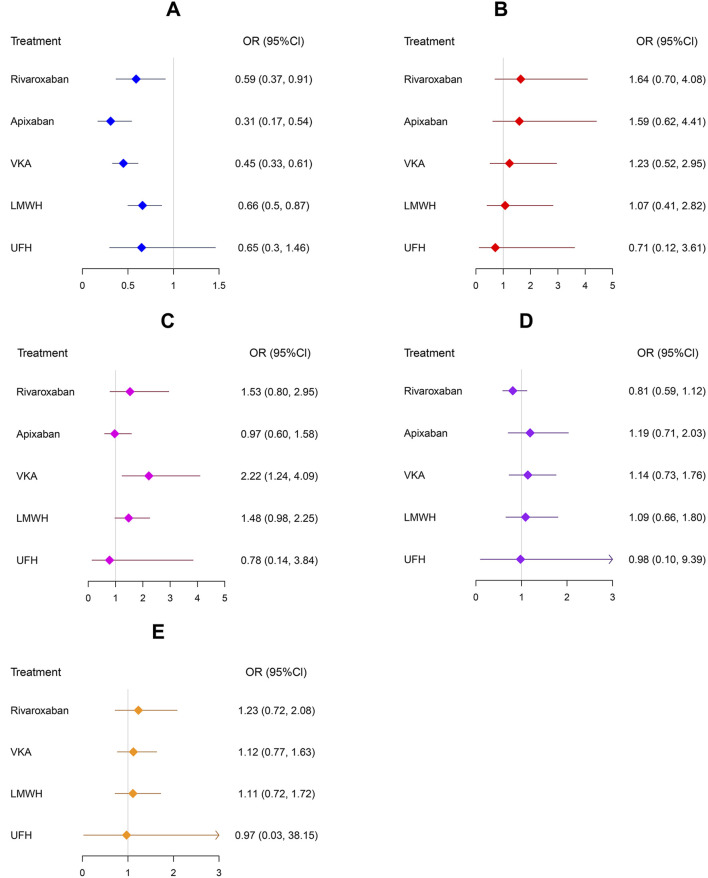
Forest plots. Crl: credible interval; DOACs: direct oral anticoagulant; LMWH: low molecular weight heparin; OR: odds ratio; UFH: unfractionated heparin; VKA: Vitamin K Antagonist. **(A)** CRT. **(B)** Major bleeding. **(C)** Bleeding. **(D)** All-cause mortality. **(E)** Adverse events.

**FIGURE 7:**
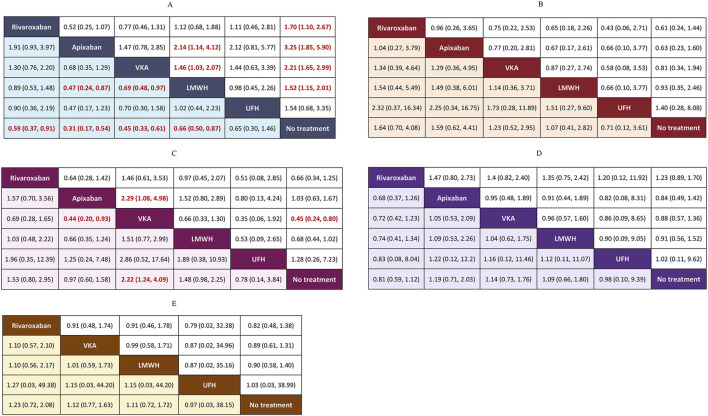
League tables of outcome analyses. Effect sizes are presented as OR of means with 95% Crl. Figure should be read from left to right, OR<1 favour the column-deffning interventions and means that the interventions in the column is associated with lower risk for the outcome than the interventions in the row. To obtain the reverse comparison OR value, reciprocals should be taken. Significant results are indicated by red bold font. Crl: credible interval; DOACs: direct oral anticoagulant; LMWH: low molecular weight heparin; OR: odds ratio; UFH: unfractionated heparin; VKA: Vitamin K Antagonist. **(A)** CRT. **(B)** Major bleeding. **(C)** Bleeding. **(D)** All-cause mortality. **(E)** Adverse events.

The results from the forest plots (Figure 6) and the league tables of outcome analyses (Figure 7) show that: 1) CRT: Compared with No treatment, Apixaban, VKA, Rivaroxaban, and LMWH significantly reduced the incidence of CRT. 95% CIs effect size ORs were 0.31 (0.17, 0.54), 0.45 (0.33, 0.61), 0.59 (0.37, 0.91), and 0.66 (0.50, 0.87), respectively, with statistically significant differences. This indicates that Apixaban, VKA, Rivaroxaban, and LMWH are significantly more effective in preventing CRT compared to No treatment. 2) Bleeding: VKA significantly increased the bleeding rate compared with Apixaban and No treatment, with ORs of 2.29 (1.08, 4.98) and 2.21 (1.24, 4.08), respectively, with statistically significant differences. This indicates that VKA is associated with a significantly higher risk of bleeding compared to Apixaban and No treatment. The other interventions did not significantly increase the incidence of bleeding events. 3) Major bleeding, all-cause mortality, and adverse reactions: No significant statistical differences were observed between the interventions and No treatment for major bleeding, all-cause mortality, or adverse reactions. Nor were there any statistically significant differences between these interventions in pairwise comparisons. Collectively, these results confirm that these interventions did not lead to a significant increase in the incidence of the above safety events.

#### 3.5.4 Ranking of network meta-analysis results

**FIGURE 8:**
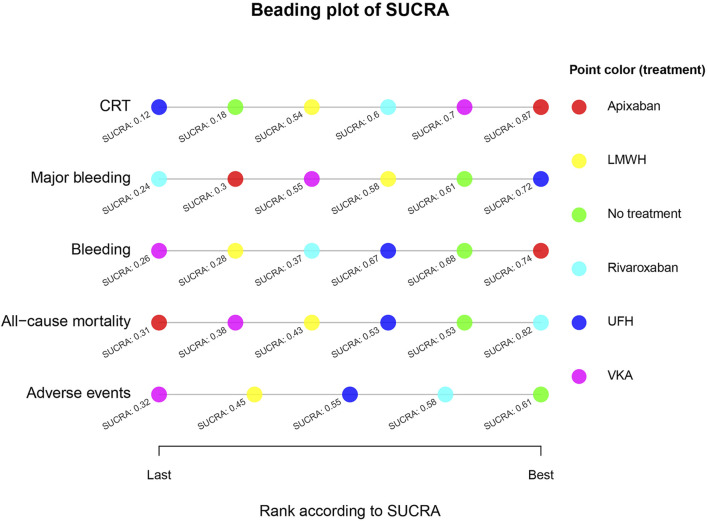
Cumulative ranking plot. For each outcome, interventions are ranked from left (worst) to right (best) according to their SUCRA values. Higher SUCRA values indicate a higher probability of being the optimal intervention for the corresponding outcome. Crl: credible interval; DOACs: direct oral anticoagulant; LMWH: low molecular weight heparin; OR: odds ratio; UFH: unfractionated heparin; VKA: Vitamin K Antagonist.

The ranking of each intervention was visualized using the cumulative ranking plot (Figure 8). The SUCRA values closer to 1 represent better performance, and those closer to 0 represent poorer performance. Based on the SUCRA values, the ranking of interventions for each outcome is as follows: (1) CRT: Apixaban > VKA > Rivaroxaban > LMWH > No treatment > UFH. (2) Major bleeding: UFH > No treatment > LMWH > VKA > Apixaban > Rivaroxaban. (3) Bleeding: Apixaban > No treatment > UFH > Rivaroxaban > LMWH > VKA. (4) All-cause mortality: Rivaroxaban > No treatment > UFH > LMWH > VKA > Apixaban. (5) Adverse reactions: No treatment > Rivaroxaban > UFH > LMWH > VKA.

#### 3.5.5 Sensitivity analysis

To ensure the robustness of results and reduce potential confounding bias, sensitivity analysis was conducted separately among high-quality RCTs. After excluding non-randomized studies, UFH was represented by only a single study, resulting in excessive statistical uncertainty and precluding valid network meta-analysis; thus, UFH was excluded from the subgroup analysis. Results showed that the findings of this network meta-analysis were generally robust. As shown in Supplementary Tables S10–S14, results were consistent except for one additional statistically significant outcome. Apixaban was significantly superior to rivaroxaban in reducing the incidence of VTE 0.47 (0.23, 0.93).

#### 3.5.6 Publication bias

The publication bias analysis results are presented in the funnel plot (Supplementary Figure S19), indicating that there may be a little heterogeneity in CRT, bleeding and adverse events outcome indicators, while the funnel plot of other indicators was symmetrical. The overall risk of publication bias was low.

## 4 Discussion

This study systematically reviewed the research trends, collaboration networks, and academic hotspots in the field of CVC-related thrombosis prevention and treatment in cancer patients using bibliometric methods. Based on 680 articles selected from the Web of Science database, statistical and visualization analyses were conducted to display the authoritative countries, institutions, and authors in this field, revealing the global development trends, current status, and research hotspots, with the aim of providing valuable insights for future research directions and clinical practice. Additionally, Bayesian network meta-analysis was employed to systematically evaluate the efficacy and safety of 5 anticoagulation regimens (rivaroxaban, apixaban, LMWH, UFH, VKA) compared with No treatment for the prevention of CVC-related thrombosis.

### 4.1 Bibliometric overview of the field

Since 2000, there have been four peaks in annual publication volume in this field, which may be related to the iteration of anticoagulation therapy technologies and updates to clinical guidelines. The early growth in publications may be associated with the accumulation of evidence supporting LMWH in the prevention and treatment of cancer-related thrombosis, while the later increase in publications is likely related to the advent of DOACs and the improvement of clinical trials. Global publication trends show that the United States holds an absolute advantage, ranking first in both publication volume and citation count, leading the clinical research and guideline development in this field, reflecting its well-established cancer diagnosis and treatment system and research platforms. The United Kingdom has the highest international collaboration rate, indicating its mature multinational and multicenter collaboration systems. Italy and China ranked second and third in publication volume, respectively.

This study provides a comprehensive analysis of the institutions and major author teams involved in the research on CVC-related thrombosis prevention and treatment in cancer patients. The core institution in this field is McMaster University in Canada, highlighting its strengths in the cross-disciplinary platform of thrombosis and oncology. The renowned scholar Philippe Debourdeau and his team have primarily focused on the development of CVC thrombosis risk prediction models (such as optimizing the Khorana score model). Over the past 5 years, their outstanding research achievements have established their strong authority and influence in this field. In terms of academic influence, this study revealed the core academic categories and distribution of academic impact in the field through journal and literature analysis. The journal analysis showed that the most publications in this field are found in hematology and oncology journals. Cochrane Database of Systematic Reviews emerged as the most influential journal in the field. The journal dual-map overlay analysis also revealed the trend of multidisciplinary collaborative research in the field of CVC-related thrombosis and anticoagulation. Highly cited articles are mainly focused on risk assessment and prevention strategies for CVC-related thrombosis, providing significant theoretical and practical guidance for the development of clinical guidelines and practice.

### 4.2 Research hotspots and frontier directions in bibliometric analysis

This study delves into keyword analysis to identify the research themes in the field of CVC-related thrombosis prevention and treatment in cancer patients, accurately capturing the frontier directions and the evolution of research topics. These insights are intended to provide ideas for researchers to conduct innovative and forward-looking studies. The results indicate that early research in this field focused on the study of initial technologies and drugs, with keywords such as “Hickman,” “Ports,” and the traditional anticoagulant “Warfarin” being prominent, and the research approach primarily consisted of randomized controlled trials. As technologies have advanced and new drugs have been developed and marketed, recent research has shifted towards the development of new technologies and medications. Keywords such as “PICC”, “Rivaroxaban,” and “Meta-analysis” have emerged, and the research approach has moved towards comparing new drugs and integrating evidence-based data, which has also driven updates to clinical guidelines. Furthermore, with the introduction of “Rivaroxaban,” the issue of “safety” has become a core focus for researchers, as balancing the benefits of anticoagulation with the risk of bleeding is crucial in clinical practice.

At present, the formation of CVC-related thrombosis in cancer patients is the result of multiple interacting factors, mainly involving patient-related factors, catheter-related factors, and tumor-related factors (Bray et al., 2018; Wang et al., 2016).

With the continuous advancement of research, anticoagulant therapies used after the occurrence of CVC-related thrombosis in cancer patients have evolved from warfarin, which has a narrow therapeutic window and requires frequent monitoring, to LMWH, which inhibits coagulation factors Xa and IIa, is administered at fixed doses, does not require routine monitoring, and has become a first-line option, and further to DOACs, such as rivaroxaban, which directly inhibits coagulation factor Xa or IIa, allows oral administration, has a rapid onset of action, and improves treatment convenience and patient adherence. A considerable number of studies have shown comparable efficacy between LMWH and DOACs in the prevention and treatment of CVC-related thrombosis in cancer patients. Jiaxuan Xu et al. (Xu et al., 2023) conducted a retrospective analysis of 217 cancer patients who developed thrombosis after PICC placement and were treated with rivaroxaban or LMWH; the results showed similar recurrence rates between the two groups (2.0% vs. 1.7%), while non-major bleeding events within 180 days were higher in the rivaroxaban group than in the LMWH group (13.1% vs. 5.1%). The continuous evolution of anticoagulant therapies has provided more options for individualized treatment and promoted the precision management of thrombosis prevention in cancer patients. Meanwhile, CVC technology has also evolved from infusion ports and Hickman catheters to PICCs. Hickman catheters increase the risk of infection and thrombosis due to external exposure; infusion ports are fully implanted subcutaneously with a lower infection risk but require surgical procedures and are costly; PICCs, inserted via peripheral veins, offer simpler operation, minimal trauma, and long-term indwelling capability, significantly reducing complication risks. Furthermore, continuous optimization of PICC materials and design has improved safety and convenience, enhanced patient quality of life, and provided important support for the long-term management of cancer treatment.

Based on the results of this study, LMWH, as the longest-standing research theme, has accumulated sufficient evidence from clinical studies and is currently the first-line drug for thrombus prevention in clinical practice. “Risk factors” and “prevention” have emerged as high-frequency research themes, reflecting the academic community’s heightened attention to the risk of CVC-related thrombosis and preventive strategies in cancer patients, which holds significant research value. If DOACs, such as rivaroxaban, can make breakthroughs in safety and efficacy, they will not only provide more evidence for consensus in related guidelines but also potentially optimize CRT prevention strategies, offering more options for clinicians. This could enhance patient survival quality and treatment adherence, which is of profound significance for improving the integrated cancer treatment system. Therefore, this study, in combination with network meta-analysis, further investigates the efficacy and safety of DOACs in preventing CVC-related thrombosis in cancer patients, aiming to provide additional evidence-based data.

### 4.3 Evaluation of each intervention in network meta-analysis

This study included 19 RCTs with a total of 5,687 patients based on strict inclusion and exclusion criteria. The results confirmed that Apixaban, VKA, Rivaroxaban, and LMWH significantly reduced the risk of CRT compared to No treatment, with the SUCRA ranking as follows: Apixaban > VKA > Rivaroxaban > LMWH. Although VKA ranked the highest in terms of efficacy and showed no significant difference in major bleeding risk compared to No treatment, its bleeding risk was significantly higher than that of No treatment, indicating that VKA increases the bleeding risk for patients, affecting medication safety. In the clinical application, VKA is represented by warfarin, and the therapeutic window of warfarin is narrow, so the anticoagulant effect needs to be judged by blood test International Normalized Ratio (INR). An excessively high INR may increase the bleeding risk, while a low INR may lead to thrombosis, requiring regular monitoring and dose adjustments. Furthermore, vitamin K-rich foods can affect the efficacy of warfarin, making dietary restrictions an inconvenience for patients.

Apixaban and Rivaroxaban, as next-generation anticoagulants, are characterized by rapid onset, convenient administration, and no need for routine monitoring, making them the preferred choice for anticoagulation therapy in many cases. However, further research is needed on their application in thrombosis prevention. In this study, Apixaban ranked the highest in efficacy, with Rivaroxaban ranked third. In terms of safety, neither drug showed a significant difference compared to No treatment. The notable efficacy of DOACs is likely due to their targeted inhibition of specific coagulation factors and their higher pharmacokinetic stability, providing evidence for the clinical application of DOACs in recent years. Considering both efficacy and safety, Apixaban and Rivaroxaban maintain a good balance between the two, providing optimal efficacy for patients, making them the best choices in this study.

LMWH ranked third in efficacy, with no significant difference in safety compared to No treatment. The SUCRA values for safety were better than VKA, indicating good safety. Supported by substantial clinical trial evidence, LMWH is often the first-line drug for thrombosis prevention in clinical practice. Although subcutaneous administration may cause adverse reactions or mild discomfort, its overall safety is well recognized.

UFH ranked the lowest for CRT prevention, with efficacy similar to No treatment, indicating it did not effectively reduce the risk of CRT. However, its safety results were relatively good. Furthermore, its small sample size (n = 224) may have led to insufficient statistical power, so the results should be interpreted with caution. This study also found inconsistency between direct and indirect evidence for the UFH vs. LMWH comparison (P < 0.05), which may be due to heterogeneity in the dosing regimen and biases from small sample studies. Given that UFH is administered by injection, it has gradually been replaced in clinical practice by LMWH, which has a longer half-life and more stable anticoagulant effects.

This study used a hierarchical Bayesian model, and the convergence of the model was fully validated through trace density plots, and PSRF values (which were slightly above 1.1 initially but converged to 1 in the final iterations), indicating stable iterations and reliable posterior distributions. To address the inconsistency in closed loops, an inconsistency model was used for analysis, ensuring rigor and supporting the clinical applicability of the results. To address the inherent risk of confounding bias in non-RCTs, a sensitivity analysis was conducted among RCTs to verify the robustness of the results. Overall, the study conclusions remained generally consistent, further supporting the reliability and stability of the network meta-analysis findings. The findings suggest that Apixaban and Rivaroxaban are the best preventive anticoagulation strategies for CVC-related thrombosis, followed by LMWH. However, clinical decisions should consider individual patient assessments. According to guideline recommendations (Chinese Thrombosis Guidelines Expert Committee, 2018; Lyman et al., 2015), prophylaxis for patients at moderate to high risk of venous thromboembolism can maximize medication safety, reduce the risk of CRT, and minimize bleeding risks. The Khorana score, a risk assessment system for predicting VTE in cancer patients, can be used to evaluate these risks, as CRT risk factors may include age, chemotherapy, tumor location and stage, coagulation function, and surgery (Meng et al., 2020; Reitzel et al., 2019; Sridhar et al., 2020). All anticoagulants carry a bleeding risk, which must be carefully weighed. Wang X. et al. (2024) constructed a PICC-related venous thrombosis nomogram for lymphoma patients via a double-center cohort study, identifying 5 independent risk factors with good predictive efficacy in both training and validation sets. Another retrospective nested case-control study by Wang X. X. et al. (2024) established a CRT predictive nomogram for cancer patients containing 9 independent risk factors, which achieved high discrimination in training and validation cohorts with AUCs of 0.832 and 0.827, respectively. These CRT-specific nomogram models are more targeted than the Khorana score and provide a refined basis for individualized thromboprophylaxis in cancer patients with central venous catheters. For gastrointestinal cancer patients, who are at higher risk of gastrointestinal bleeding, Rivaroxaban should be used cautiously, and Apixaban is recommended. A large retrospective cohort study published in 2021 (Dawwas et al., 2021) assessed the efficacy and safety of Apixaban vs. Rivaroxaban in VTE patients and found that compared to Rivaroxaban, Apixaban significantly reduced the absolute risk of gastrointestinal bleeding (OR = 0.01, 95% CI: 0.01 ∼ 0.01) within 2 months. Rivaroxaban was associated with a higher risk of major gastrointestinal bleeding complications. Therefore, clinical anticoagulation decisions should carefully consider multiple factors.

### 4.4 Evidence-based support of network meta-analysis

Existing meta-analyses primarily compare two drugs or a drug with No treatment, which cannot provide a comprehensive and intuitive comparison of the effects of different anticoagulants in preventing CVC-related thrombosis. There is a lack of multidimensional analysis that systematically evaluates the efficacy differences between multiple drugs. Therefore, this study has the advantage of a comprehensive evaluation, providing more accurate and thorough clinical decision-making support. Compared to other meta-analysis results, this study is consistent with most findings, but there are some differences. For example, a meta-analysis by Wang Song (Wang et al., 2011) in 2011 showed that warfarin effectively reduced the incidence of CVC-related thrombosis in cancer patients compared to a placebo group (RR = 0.68, 95% CI: 0.52–0.89), which is consistent with the results of this study. However, that study did not include bleeding and mortality risks, which differs from the present study. Another meta-analysis by Kahale LA (Kahale et al., 2018) in 2018 found that LMWH reduced the incidence of symptomatic catheter-related thrombosis within 3 months compared to No treatment (RR = 0.43, 95% CI: 0.22–0.81), which is consistent with the findings of this study. However, VKA did not reduce the incidence of symptomatic catheter-related thrombosis within 3 months compared to No treatment (RR = 0.61, 95% CI: 0.23–1.64), which is different from the present study’s findings. These discrepancies with other studies may be related to differences in sample sizes and analysis methods. The larger sample size in this study may provide more sufficient and reliable evidence for clinical decision-making.

Regarding the current guidelines (Chinese Thrombosis Guidelines Expert Committee, 2018; Falanga et al., 2023; Lyman et al., 2015; Lyman et al., 2021) on whether prophylaxis should be implemented for CVC-related thrombosis in cancer patients, based on the results of this study, anticoagulants such as Apixaban, Rivaroxaban, and LMWH significantly improve clinical outcomes compared to No treatment, and have good safety profiles. This confirms the significant value of anticoagulation prevention strategies and emphasizes the importance of thrombosis prevention in CVC management for cancer patients, which can help improve related guidelines. In terms of prophylactic strategy selection, this study’s results confirm that DOACs are the optimal preventive strategy for CVC-related thrombosis in cancer patients. They significantly reduce the risk of CRT without increasing bleeding risk and rank first in terms of adverse reactions and all-cause mortality, receiving excellent safety evaluations. This finding not only verifies the application potential of DOACs in this field but also demonstrates their positive role in balancing efficacy and safety, providing benefits to patients and improving their prognosis. It also provides evidence for the future clinical adoption of DOACs, offering safer and more effective preventive options. Therefore, this study recommends the use of anticoagulants for thrombosis prevention in cancer patients with CVCs, with DOACs as the recommended preventive strategy. This result indicates that DOACs have significant clinical application value and academic research significance. Notably, the optimal benefit-risk ratio of DOACs verified by the network meta-analysis in this study is generally consistent with the characteristic shift of research hotspots toward DOACs identified in the bibliometric analysis. This further confirms the clinical application potential of DOACs in the prophylaxis of central venous catheter-related thrombosis in cancer patients and provides a solid empirical basis for the research hotspots identified by the bibliometric analysis.

### 4.5 Limitations

This study is the first to apply Bayesian network analysis to evaluate the safety and efficacy of different prophylactic strategies for CVC-related thrombosis in cancer patients. However, there are some limitations in this study: First, the bibliometric analysis was conducted using only the Web of Science database and was restricted to English-language publications. Therefore, relevant studies indexed in other databases (e.g., Scopus, PubMed/Medline, or Embase) and non-English literature may have been missed. This database and language restriction may lead to selection bias in the bibliometric mapping (e.g., publication trends, collaboration networks, and keyword co-occurrence). As a result, the bibliometric findings should be interpreted as reflecting WoS-indexed, English-language literature rather than the full global evidence base, which may limit the comprehensiveness and generalizability of the analysis. Among the 19 studies included in the network meta-analysis, 3 were non-randomized controlled trials. Although a sensitivity analysis restricted to RCTs was further performed to verify the robustness of the results, the inherent high risk of confounding in non-randomized studies cannot be fully eliminated. Uncontrollable confounding factors may exist in design, population selection, intervention implementation, and outcome assessment in such studies, which may still impose potential impacts on the overall effect estimates. In addition, the limited number of studies resulted in a relatively small sample size for UFH, which may bias the results of this study.

## 5 Conclusion

In this study, we systematically investigated the research status and effective preventive strategies in the field of anticoagulation for CRT in cancer patients by combining bibliometric analysis and network meta-analysis. The bibliometric analysis revealed that this field shows clear characteristics of international collaboration, with the United States leading the world in academic output and influence. The research hotspots in this field have evolved from traditional anticoagulants such as warfarin and LMWH to DOACs, while CVC technology has also undergone continuous innovations, from infusion ports and Hickman catheters to PICCs. The synergistic development and advancement in both aspects have significantly improved the prevention and treatment of CRT in cancer patients. Additionally, the combined application of bibliometric analysis and network meta-analysis in this study provides a reference for the in-depth investigation of clinical intervention strategies in other fields.

The network meta-analysis results showed that Apixaban, VKA, Rivaroxaban, and LMWH effectively reduced the risk of CRT in cancer patients compared to No treatment, but VKA has a significant risk of bleeding. In summary, DOACs showed the most favorable benefit-risk ratio. This study provides supporting evidence for the efficacy of DOACs in preventing CRT. Nevertheless, clinical evidence for DOACs remains relatively limited, and research on long-term safety data is urgently needed. In the future, additional large-scale, multicenter clinical trials are required to validate these findings.

## Data Availability

The original contributions presented in the study are included in the article/Supplementary Material, further inquiries can be directed to the corresponding authors.
