# Correction: Efficacy and safety evaluation of thromboprophylaxis strategies for central venous catheter-related thrombosis in cancer patients: a bayesian network meta-analysis and bibliometric analysis

**DOI:** 10.3389/fphar.2026.1863333

**Published:** 2026-05-04

**Authors:** Mingyu Meng, Qimeng Xu, Shiran Qin, Xiaoyu Chen, Congzhi Qin, Muling Shen, Dandan Xu, Shiyao Pang, Jingsi Zhong, Yuan Fan, Yongxia Xu, Qin Yi, Henghai Su, Jian Qin

**Affiliations:** 1 Department of Pharmacy, Guangxi Academy of Medical Sciences and the People’s Hospital of Guangxi Zhuang Autonomous Region, Nanning, China; 2 Department of Pharmacy, Guangxi Medical University, Nanning, China; 3 Department of Pharmacy, Zhuzhou Central Hospital, Zhuzhou, China; 4 Department of Radiotherapy III, Clinical Oncology Center, Guangxi Academy of Medical Sciences and the People’s Hospital of Guangxi Zhuang Autonomous Region, Nanning, China

**Keywords:** anticoagulation, bibliometrics, cancer, central venous catheter, meta-analysis

Author Jian Qin was erroneously omitted as corresponding author. The correct corresponding authors are: Jian Qin and Henghai Su.

The original article has been updated.

