# Supplementary material for: Efficacy and safety evaluation of thromboprophylaxis strategies for central venous catheter-related thrombosis in cancer patients: a bayesian network meta-analysis and bibliometric analysis
Source: Front Pharmacol. 2026 Apr 14;17:1786836. doi: 10.3389/fphar.2026.1786836 (PMC13121162; doi:10.3389/fphar.2026.1786836)
Supplement: Supplementary file 1 [file Supplementaryfile1.docx]

***Supplementary*** ***material***

**Supplementary Figures**

Supplementary Figure 1. Flow chart of bibliometrics

Supplementary Figure 2. Flow chart of the study selection process

Supplementary Figure 3. Risk of bias in RCT studies

Supplementary Figure 4. Risk of bias in Non-RCT studies

Supplementary Figure 5. Collaboration relationship

Supplementary Figure 6. keyword burst analysis

Supplementary Figure 7. Nodes-splitting analysis of primary outcomes.

Supplementary Figure 8. Nodes-splitting analysis of secondary outcomes

Supplementary Figure 9. Trace density plots of CRT

Supplementary Figure 10. Trace density plots of major bleeding

Supplementary Figure 11. Trace density plots of bleeding

Supplementary Figure 12. Trace density plots of all-cause mortality

Supplementary Figure 13. Trace density plots of adverse events

Supplementary Figure 14. Diagnostic plots: CRT

Supplementary Figure 15. Diagnostic plots: major bleeding

Supplementary Figure 16. Diagnostic plots: bleeding

Supplementary Figure 17. Diagnostic plots: all-cause mortality

Supplementary Figure 18. Diagnostic plots: adverse events

Supplementary Figure 19. Funnel plots

**Supplementary Tables**

Supplementary Table 1. PRISMA 2020 Checklist

Supplementary Table 2. Search strategy for bibliometric analysis

Supplementary Table 3. Search strategy for network meta-analysis

Supplementary Table 4. Characteristics of included studies

Supplementary Table 5. Top 10 countries in terms of publications.

Supplementary Table 6. Top 10 institutions in terms of the number of publications

Supplementary Table 7. Top 10 authors in terms of the number of publications

Supplementary Table 8. Top 10 journal in terms of the number of publications.

Supplementary Table 9. The top 10 highly cited references

**
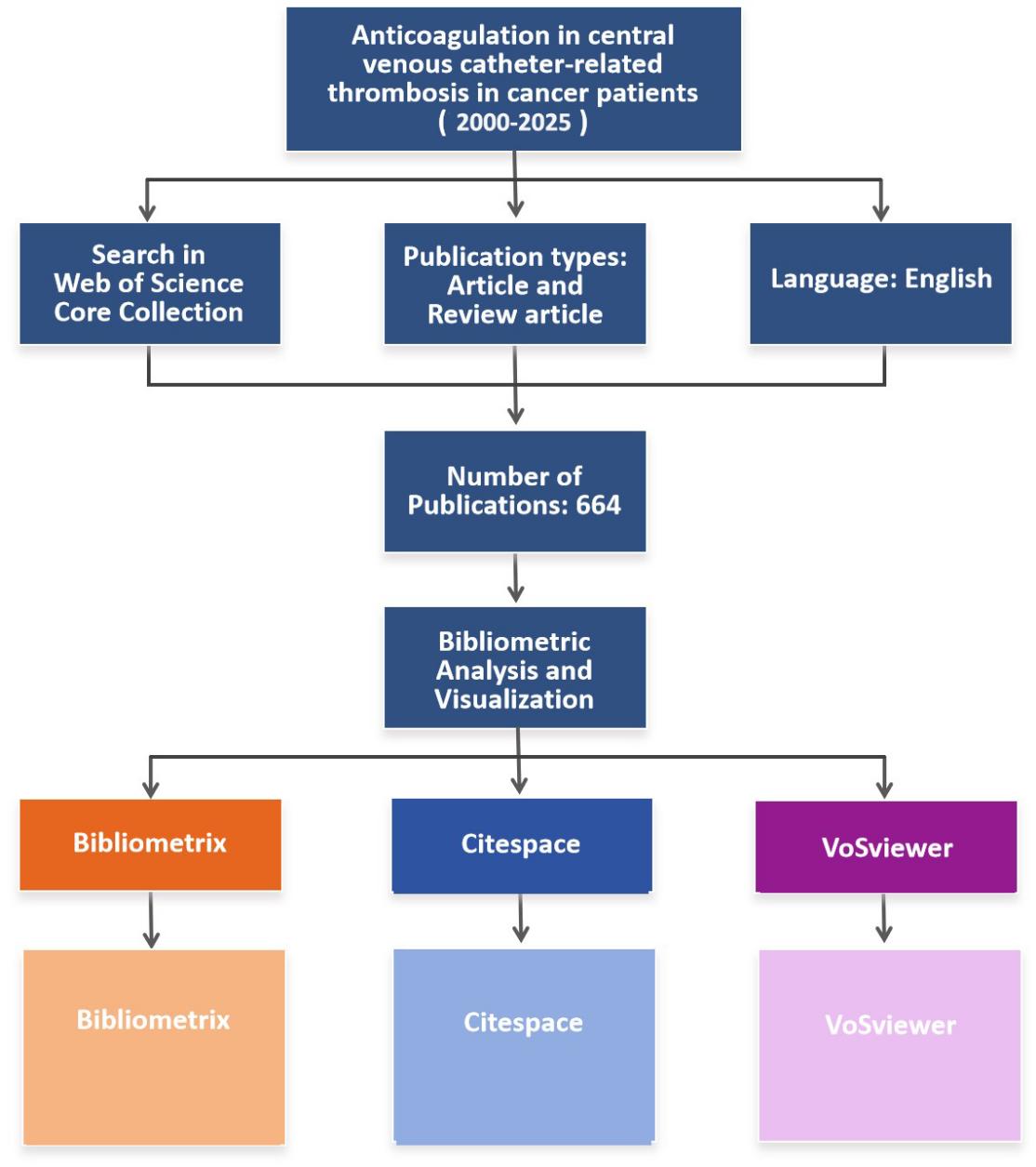
**

**Supplementary Figure 1.** Flow chart of bibliometrics

**
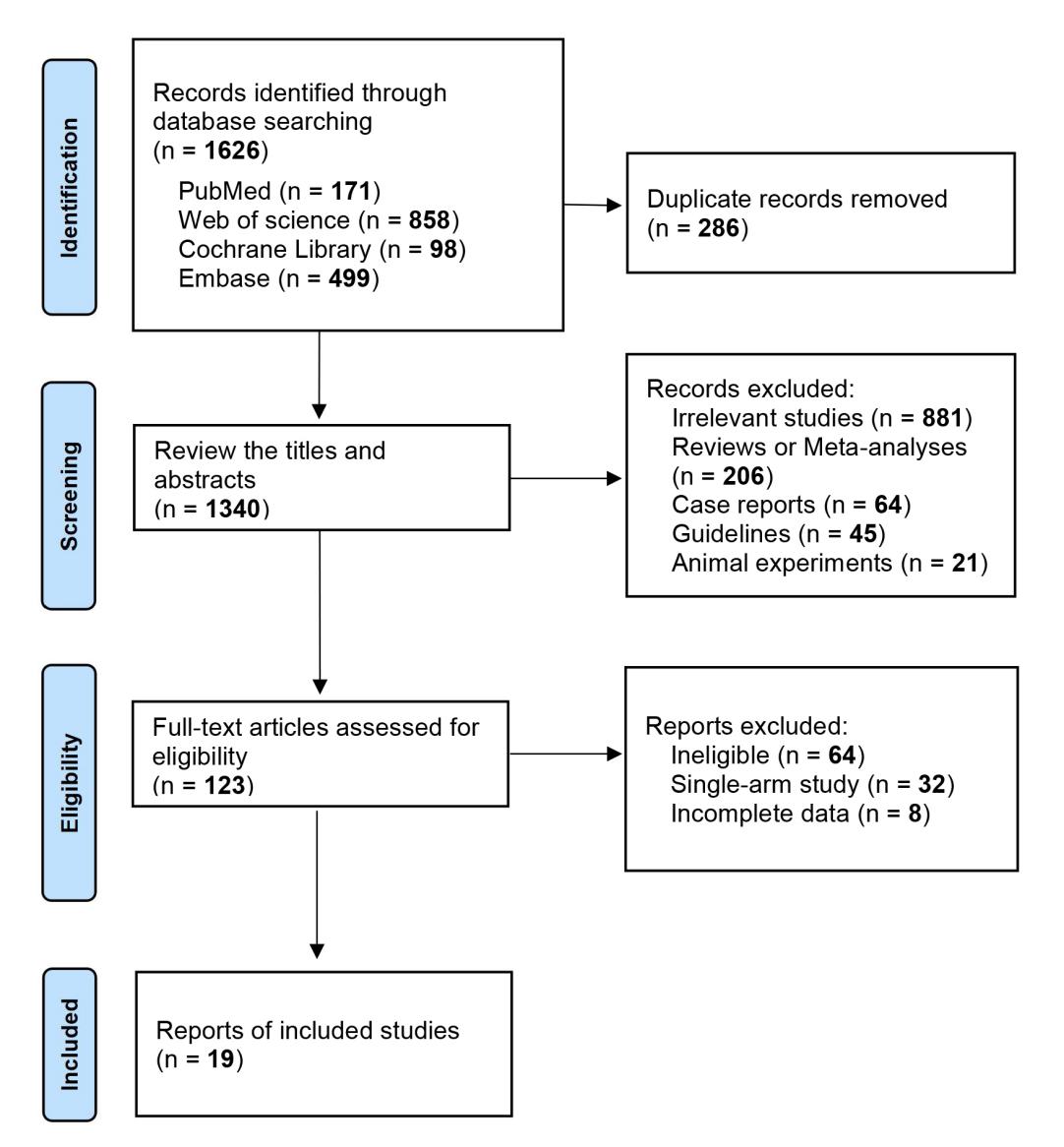
**

**Supplementary Figure 2.** Flow chart of the study selection process

**
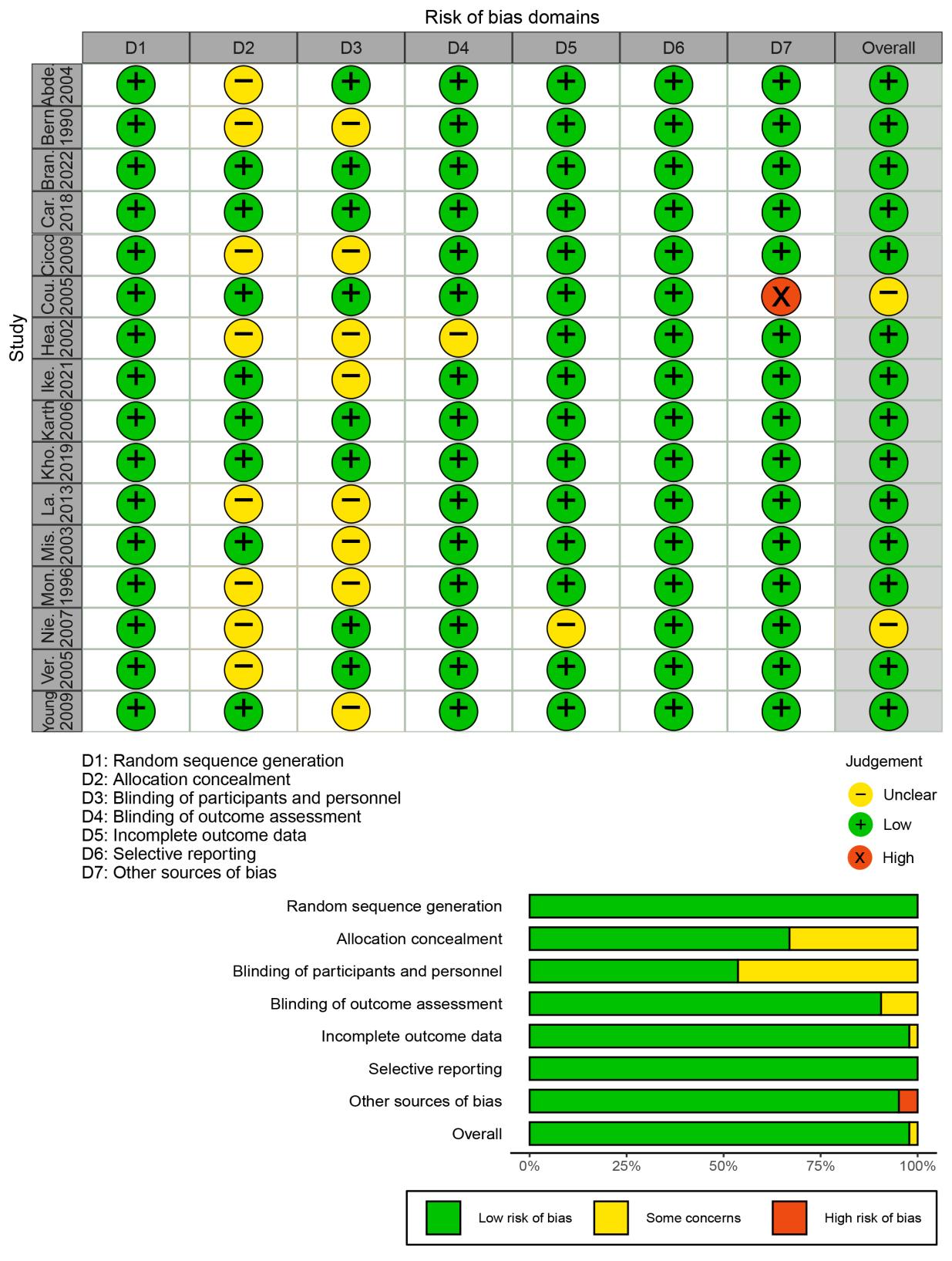
**

**Supplementary Figure 3.** Risk of bias in RCT studies


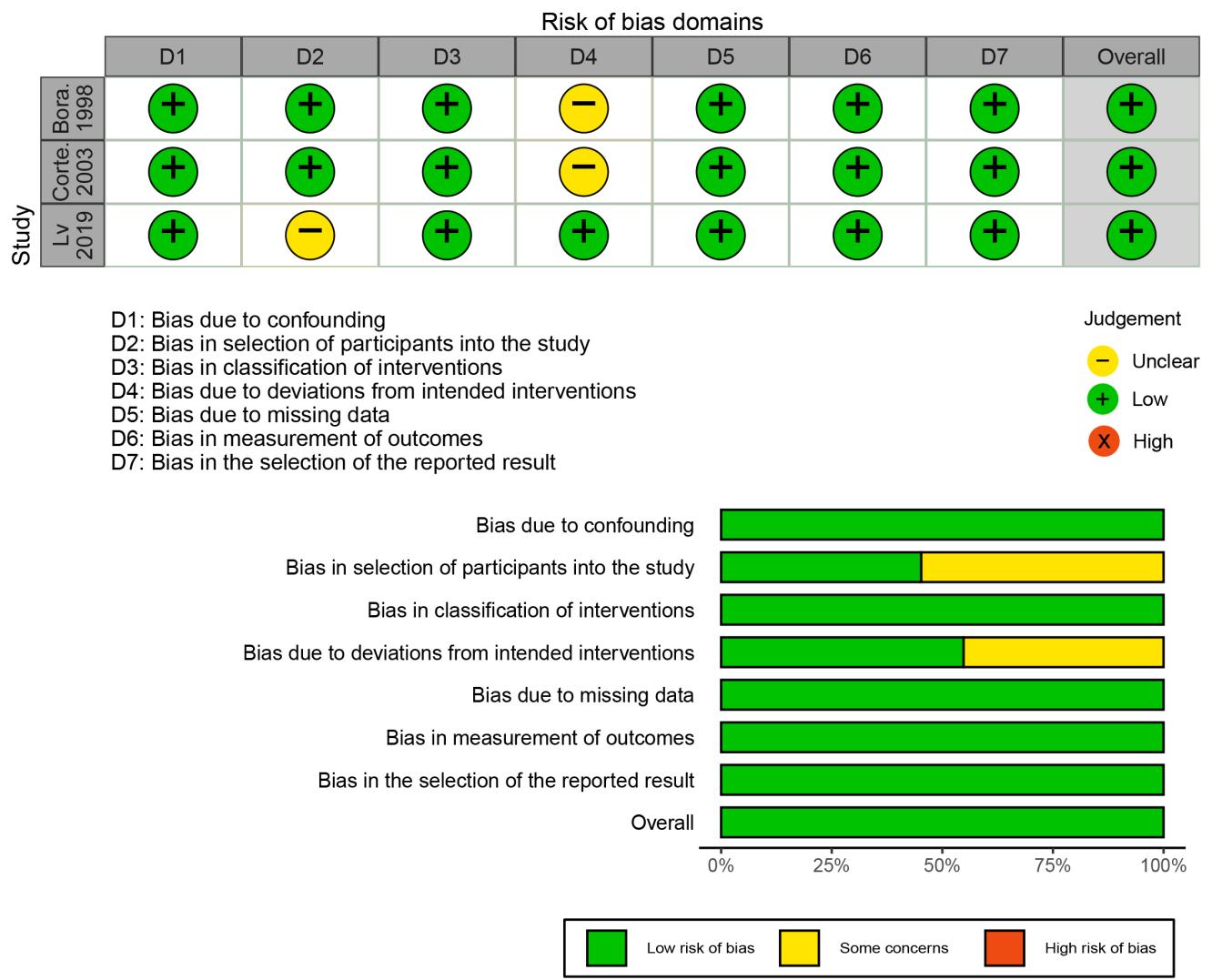


**Supplementary Figure 4.** Risk of bias in Non-RCT studies


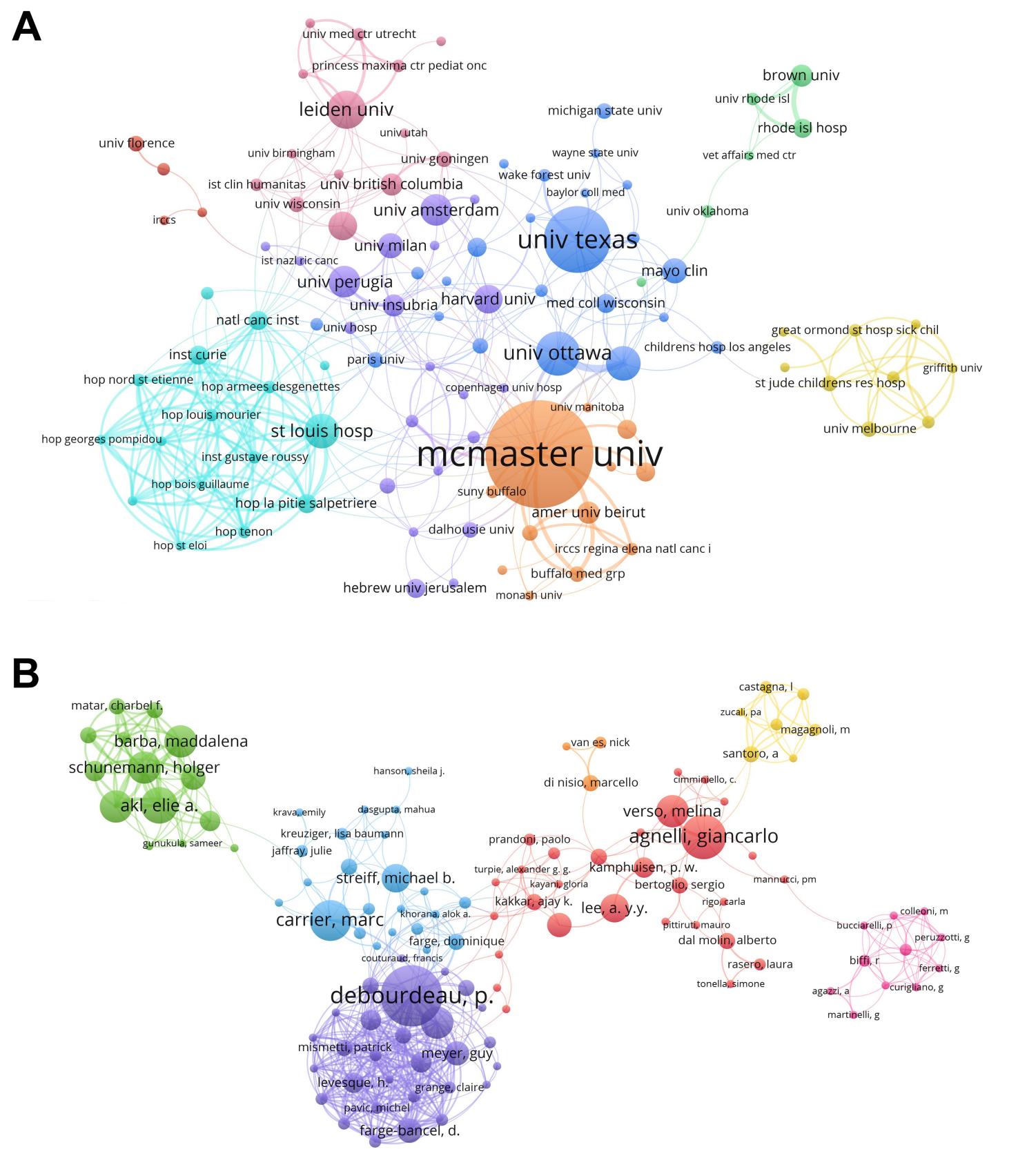


**Supplementary Figure 5.** Collaboration relationship. A: Institutional collaboration map, B: Author collaboration map.

**Note:** The thickness of the lines reflects the intensity of collaboration frequency. Based on the average year of collaboration, the score values are used for color mapping, ranging from dark blue to yellow, which represents the initiation of collaborative relationships from the earliest to the most recent.

**
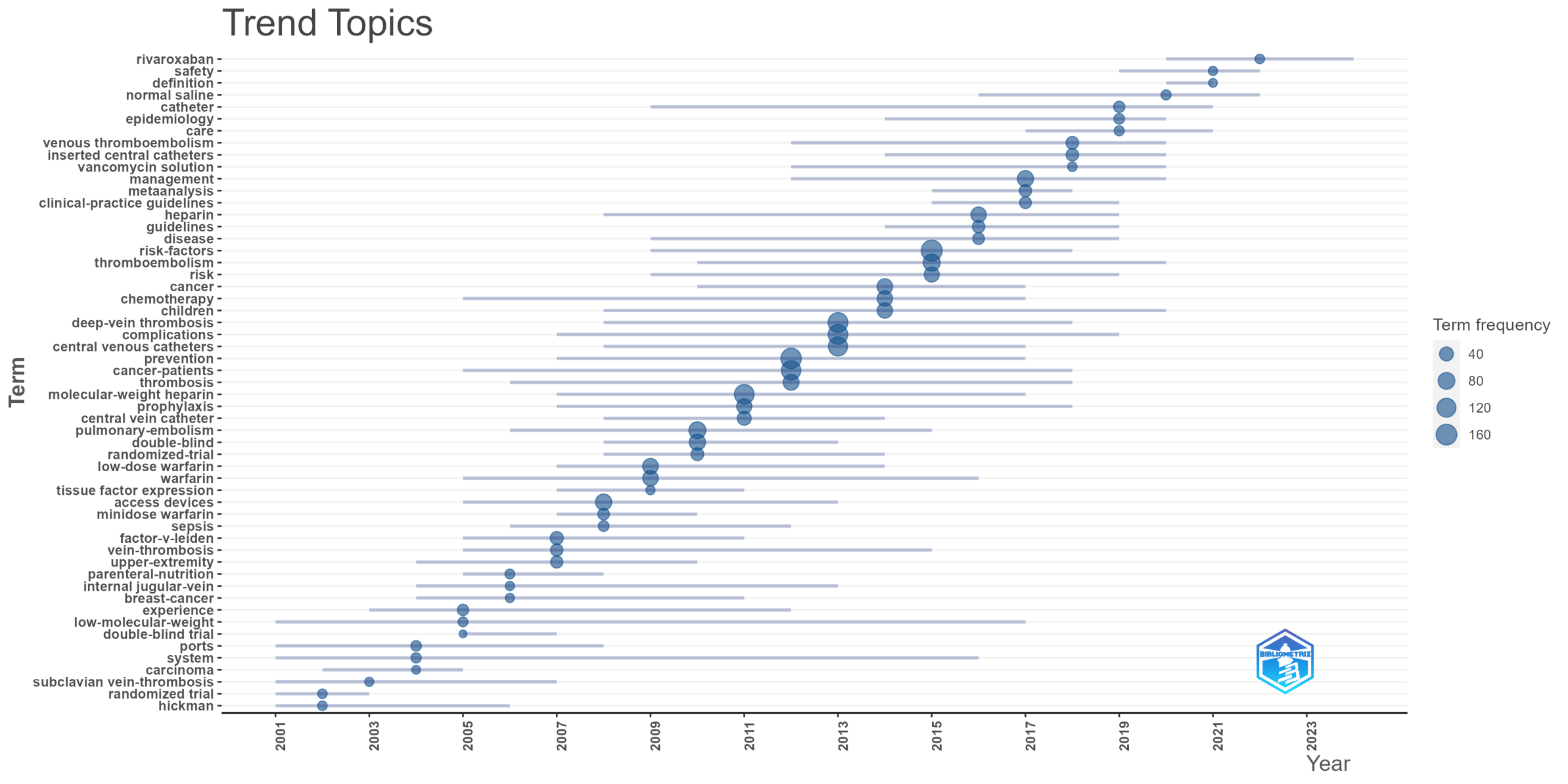
**

**Supplementary Figure 6.** keyword burst analysis.

**A CRT**


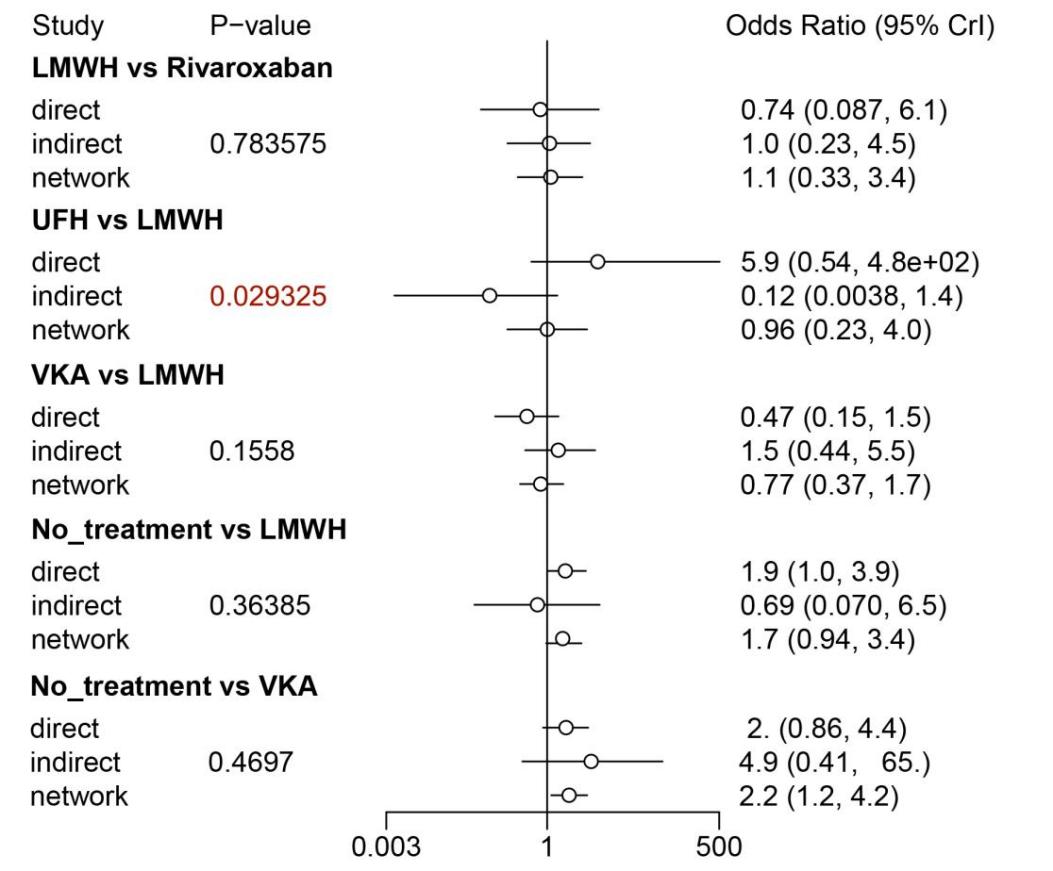
**B Major bleeding**


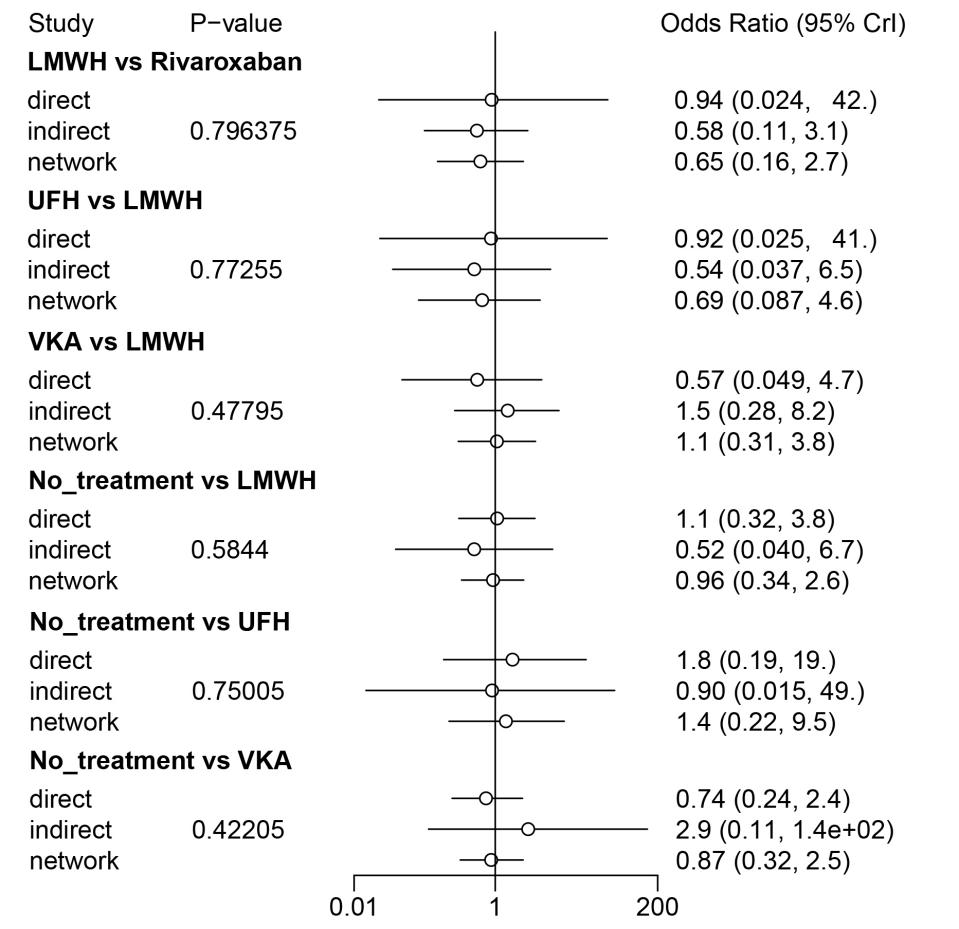


**Supplementary Figure 7.** Nodes-splitting analysis of primary outcomes.

**Note:** P > 0.05 indicates that there is no significant inconsistency which means the direct, indirect and network comparison is consistent.

**C Bleeding**

**
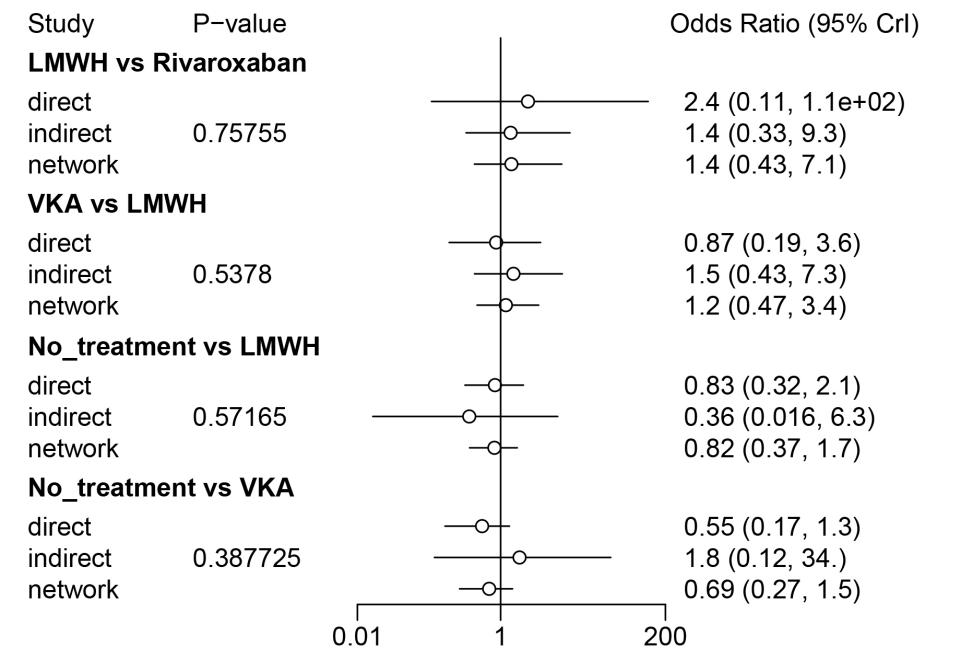
**
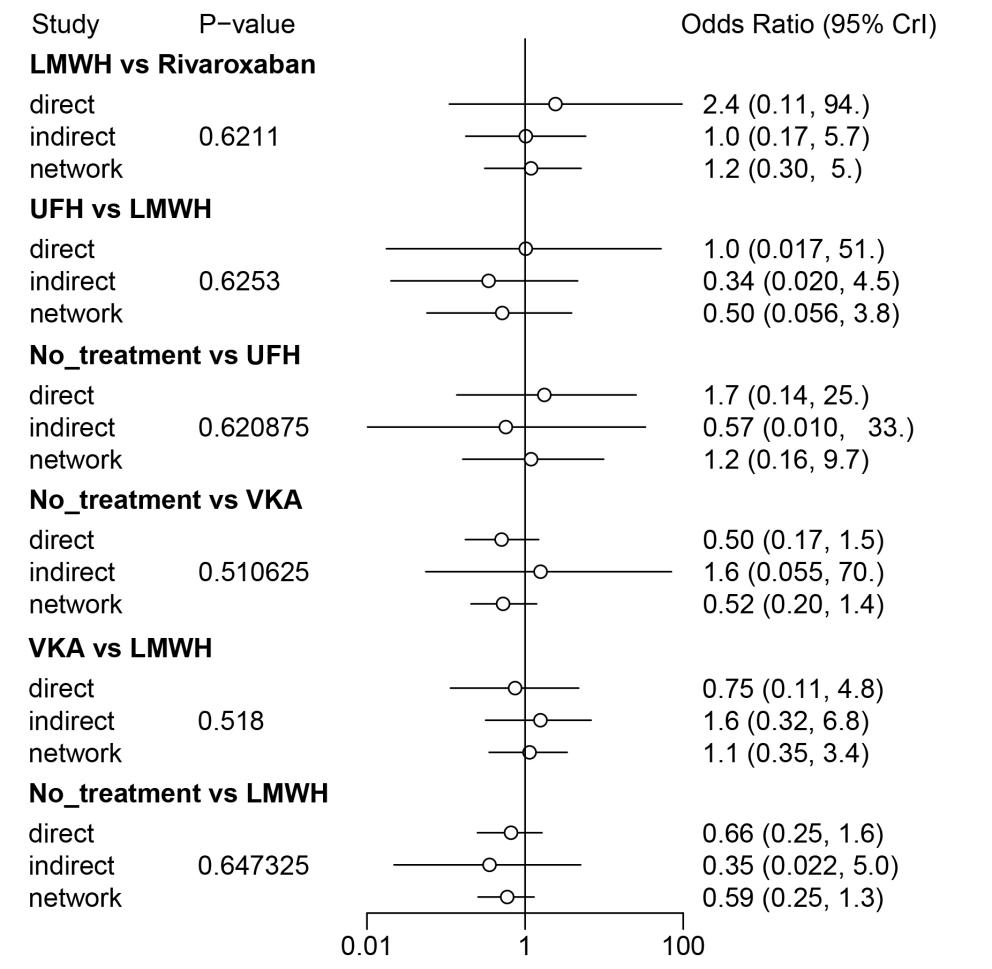
**D All-cause mortality**

**E Adverse events**

**
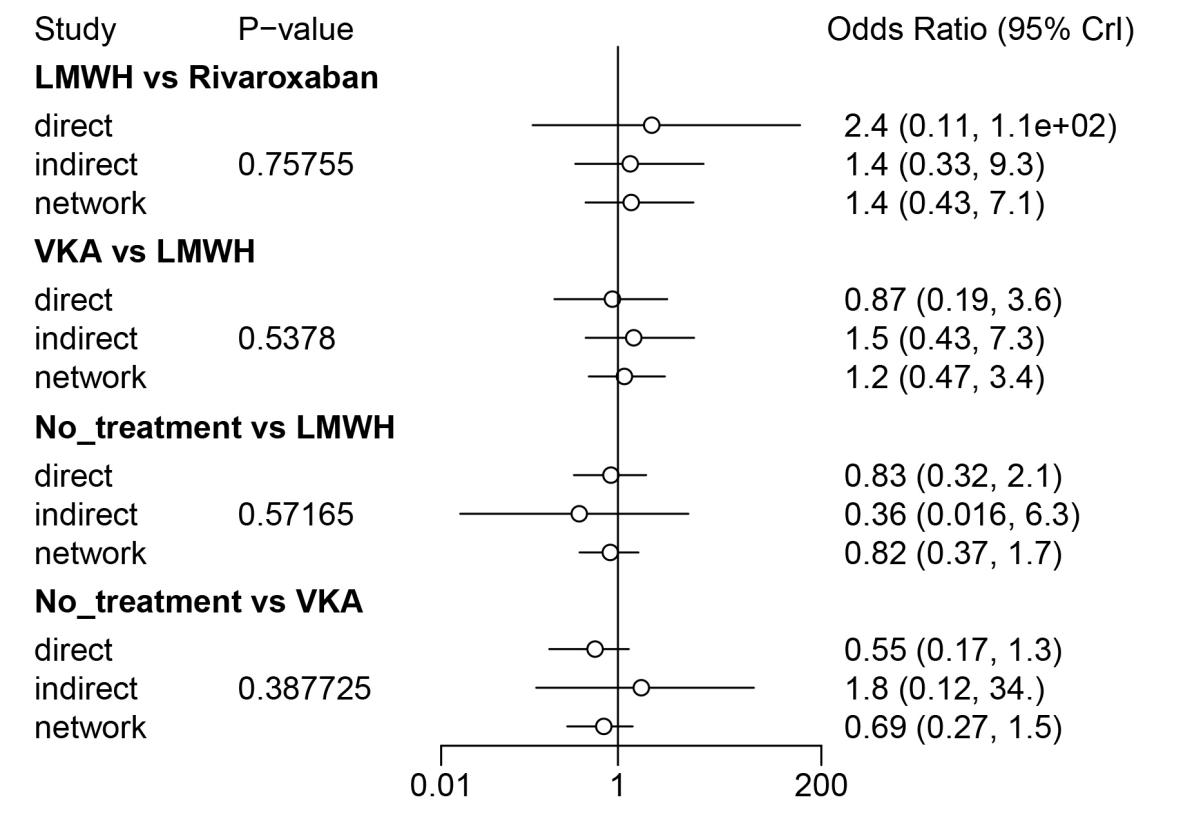
**

**Supplementary Figure 8.** Nodes-splitting analysis of secondary outcomes.

**Note:** P > 0.05 indicates that there is no significant inconsistency which means the direct, indirect and network comparison is consistent.

**
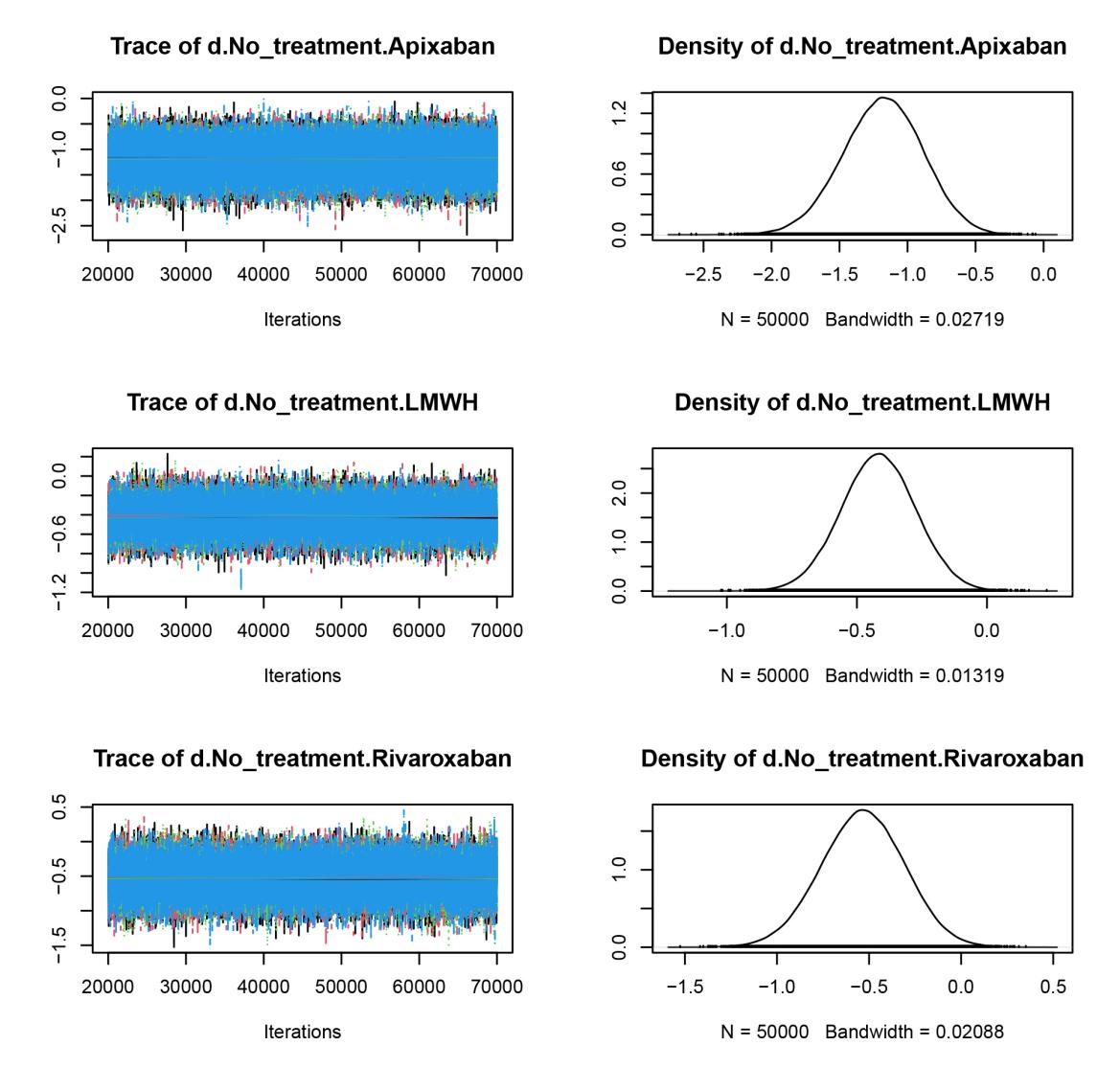
**

**
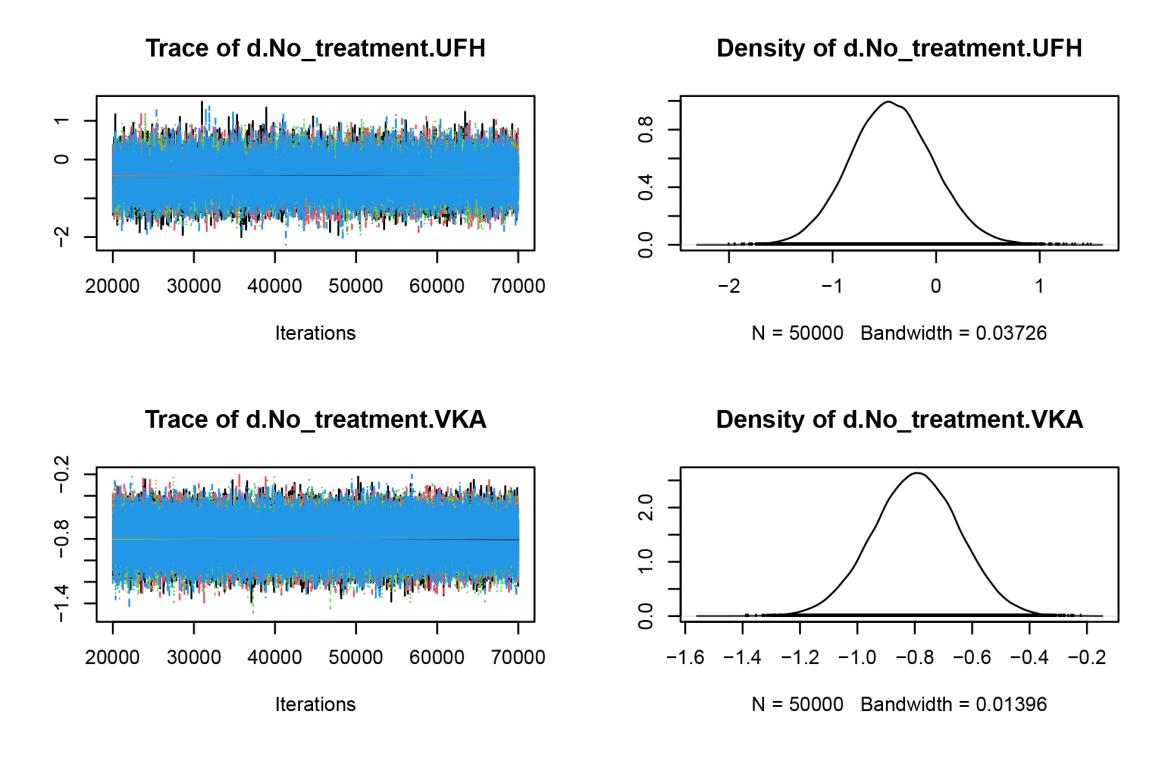
**

**Supplementary Figure 9.** Trace density plots of CRT.

**
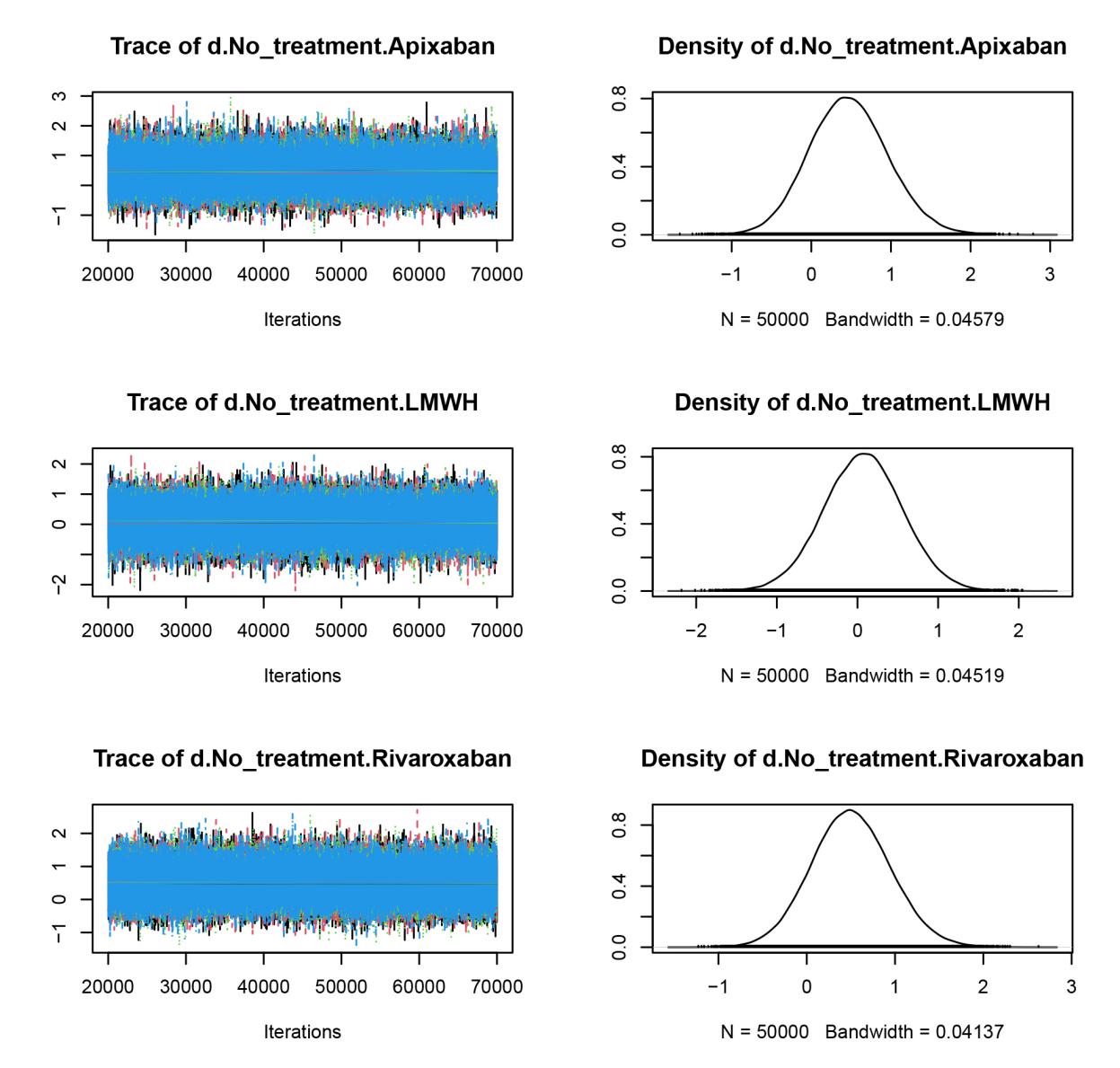
**

**Supplementary Figure 10.** Trace density plots of major bleeding.

**
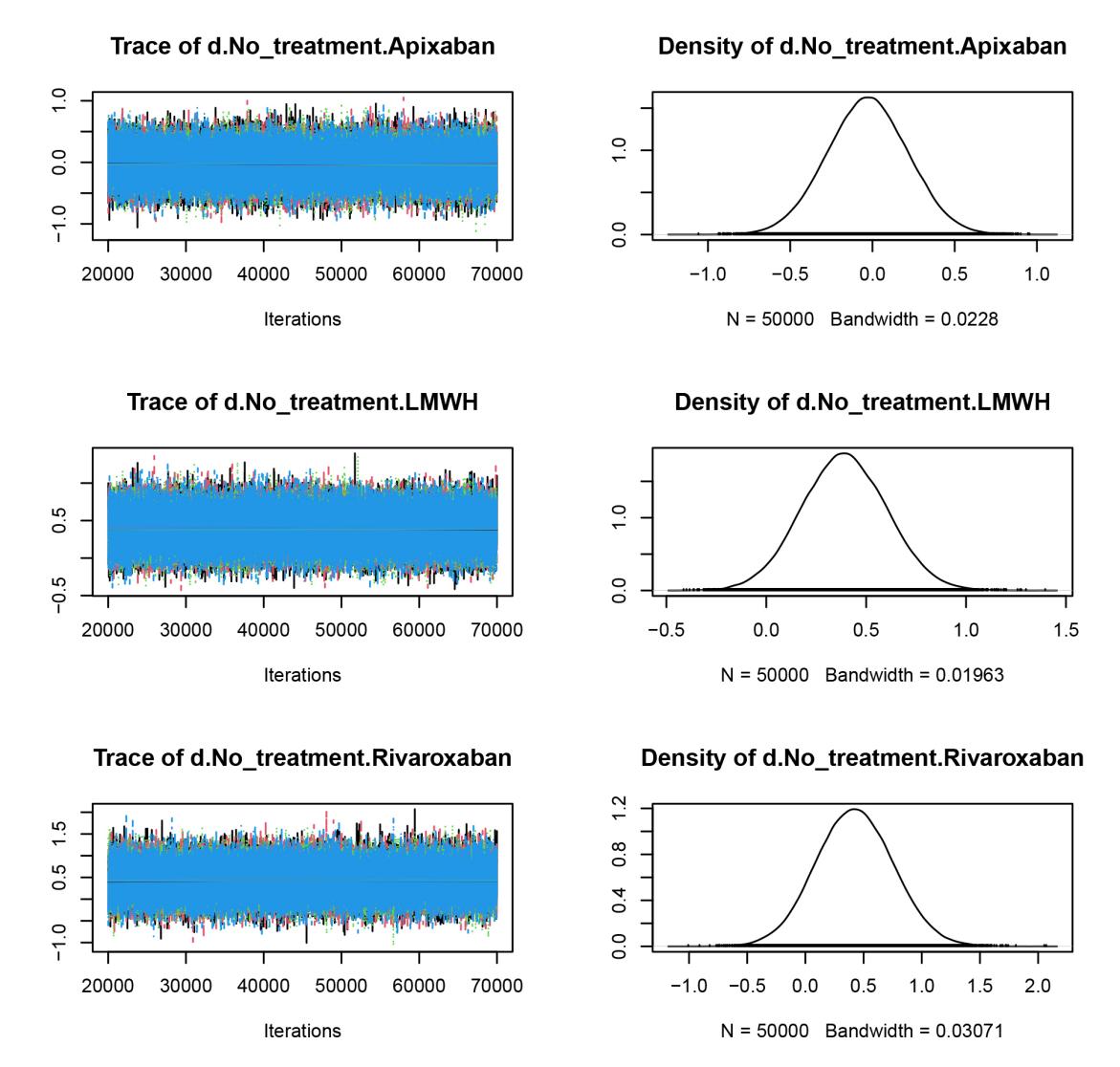
**

**
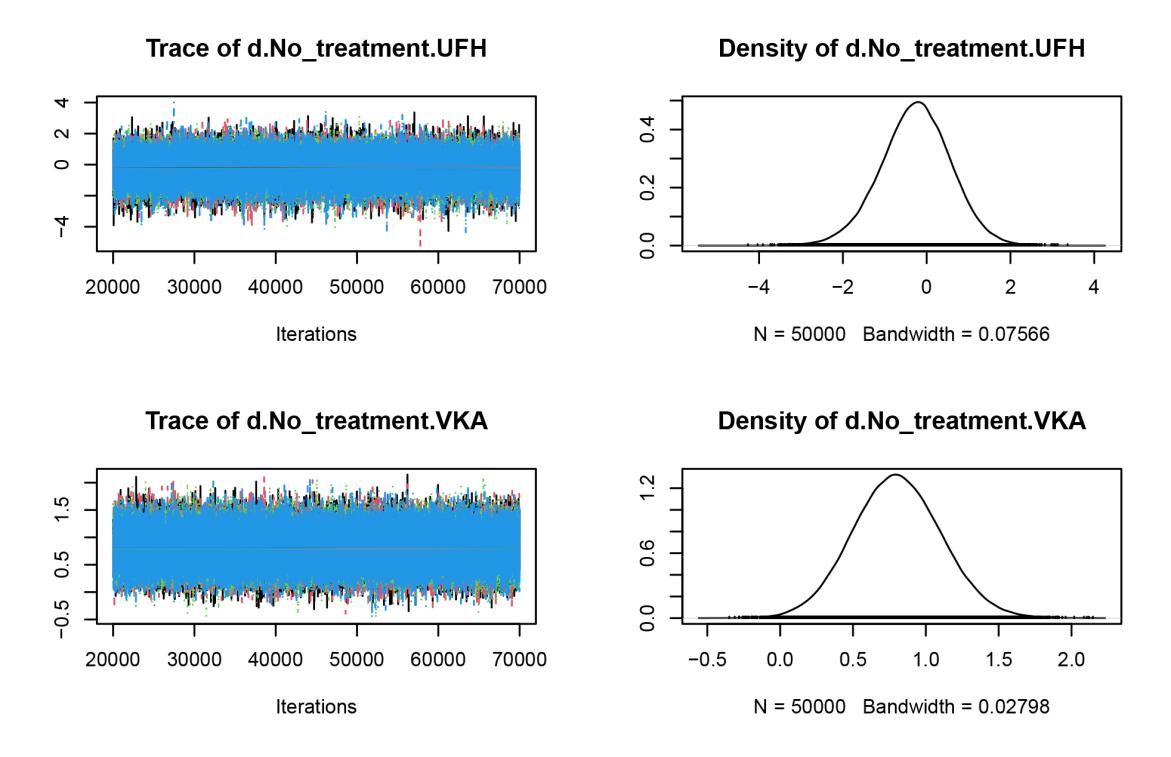
**

**Supplementary Figure 11.** Trace density plots of bleeding.

**
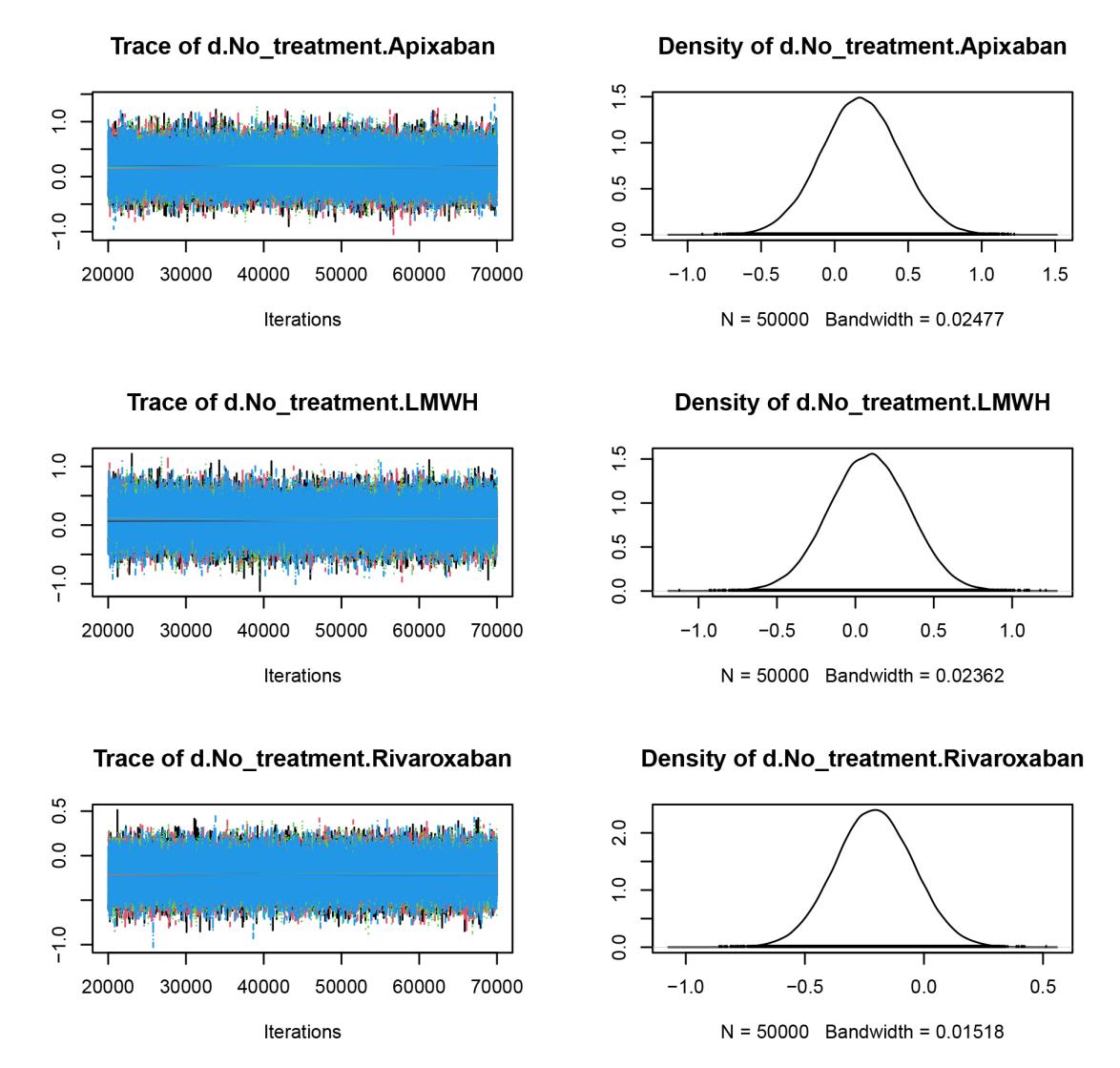
**

**
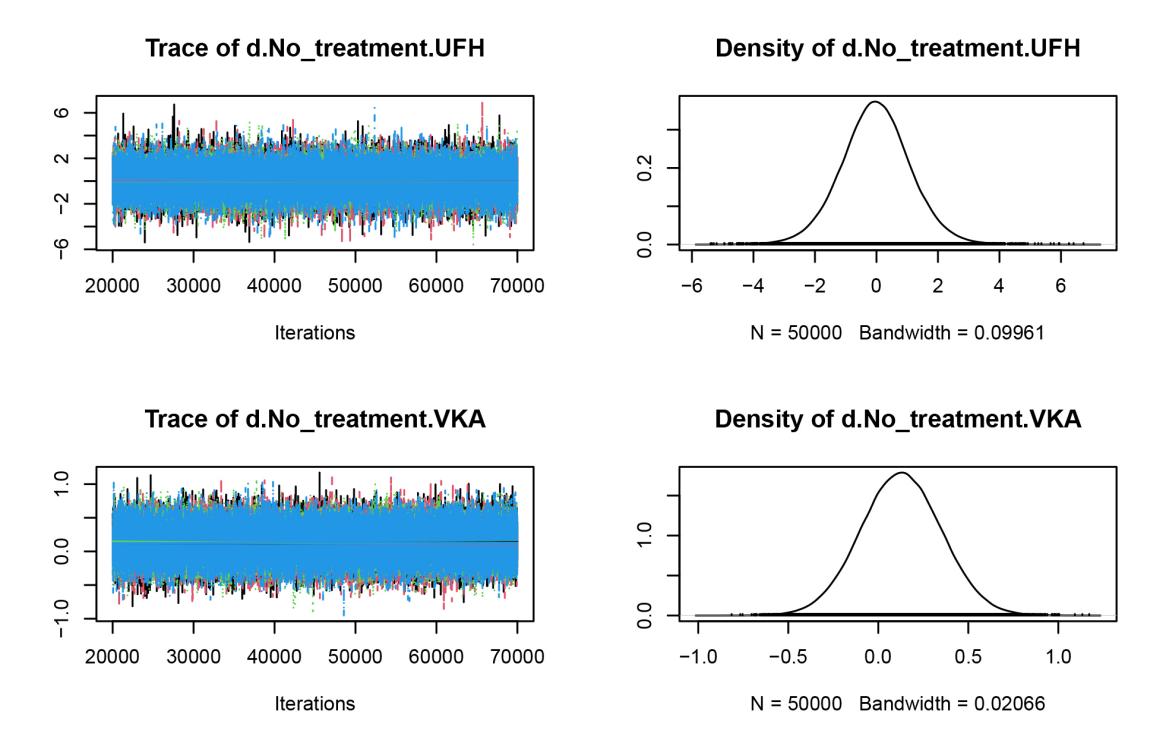
**

**Supplementary Figure 12.** Trace density plots of all-cause mortality.

**
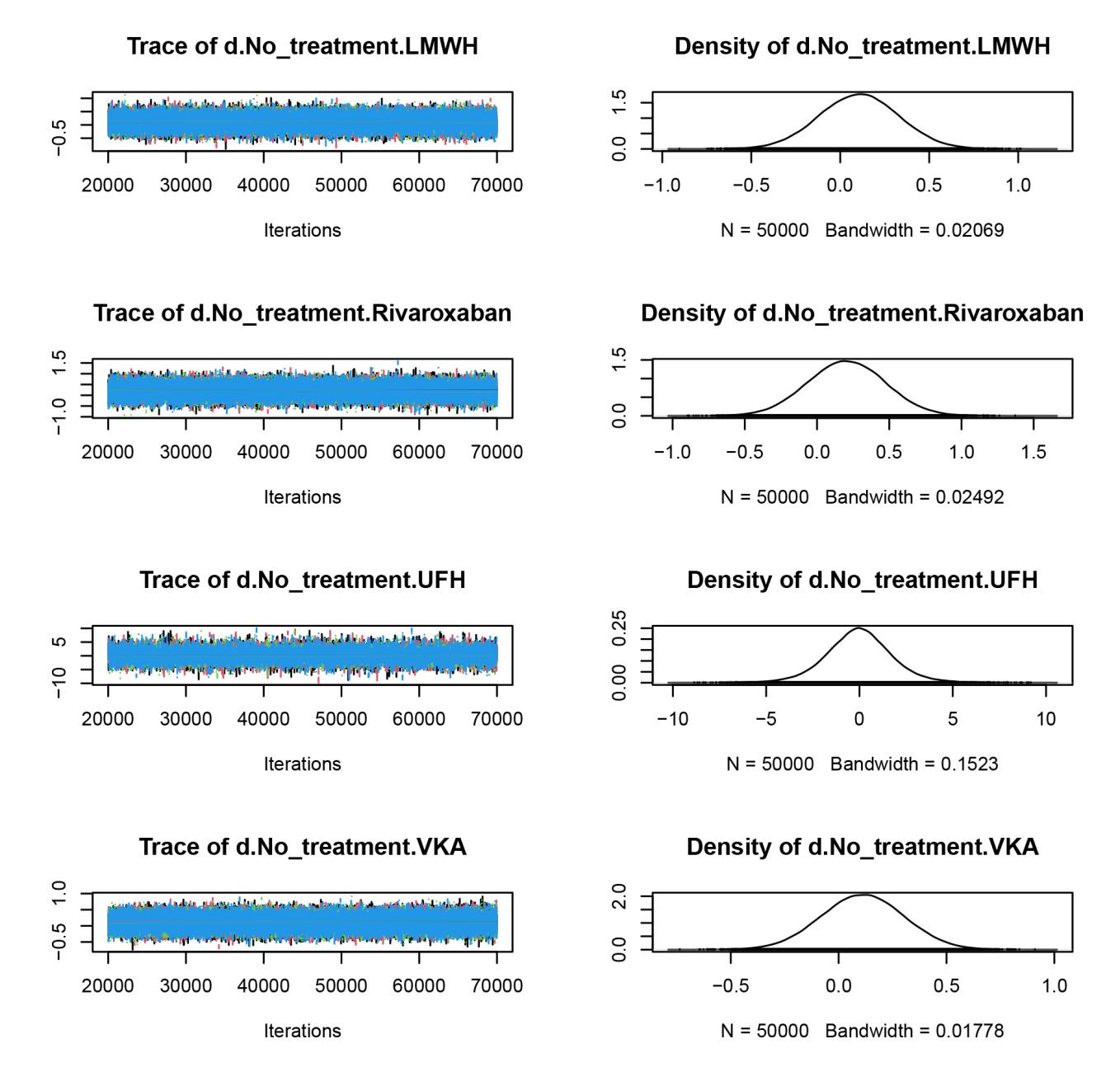
**

**Supplementary Figure 13.** Trace density plots of adverse events.

**
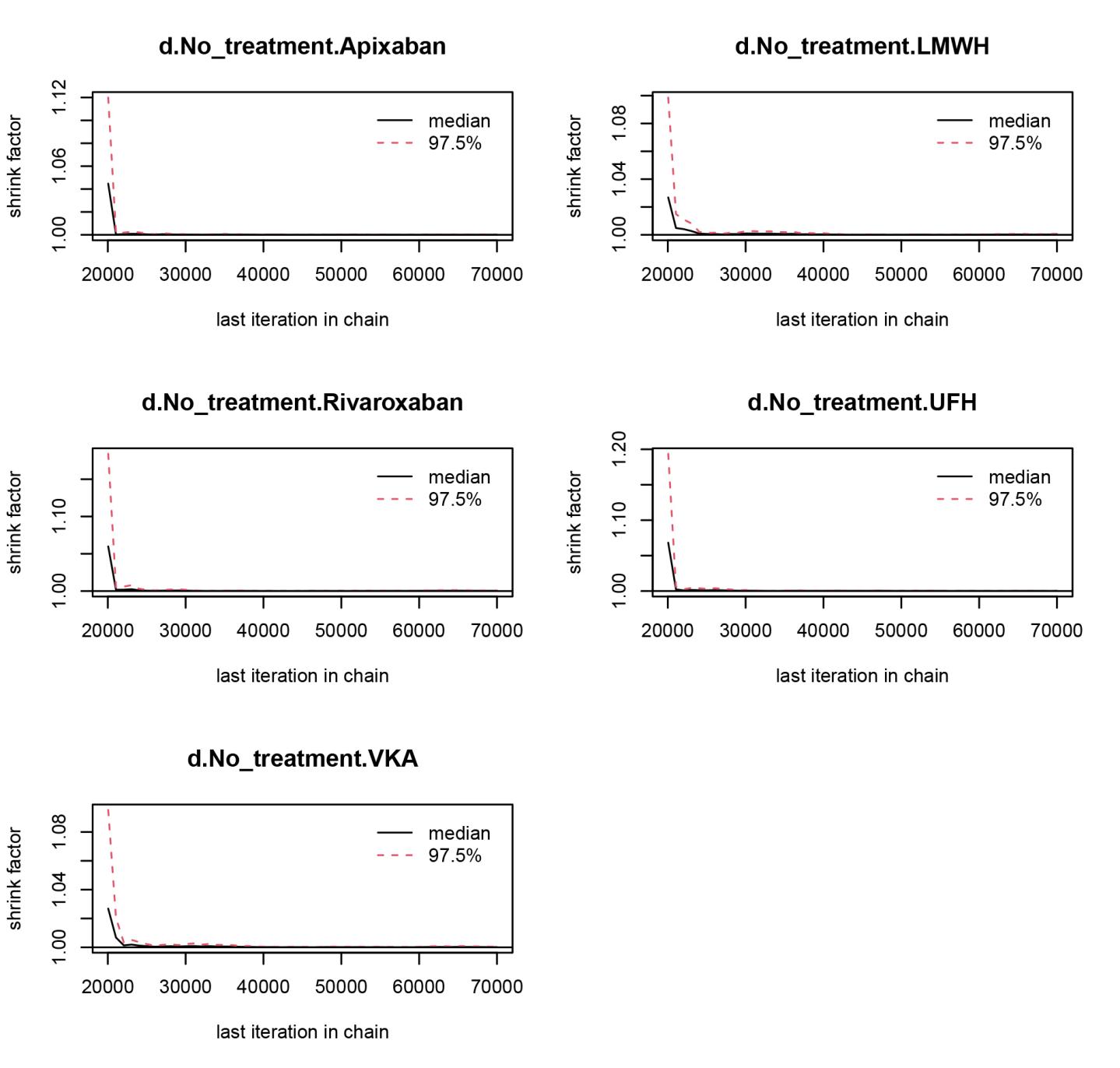
**

**Supplementary Figure 14.** Diagnostic plots: CRT. The shrink factor is the PSRF value, and the closer it is to 1, the better the convergence is, and the model is considered to have good stability and reliable results.

**
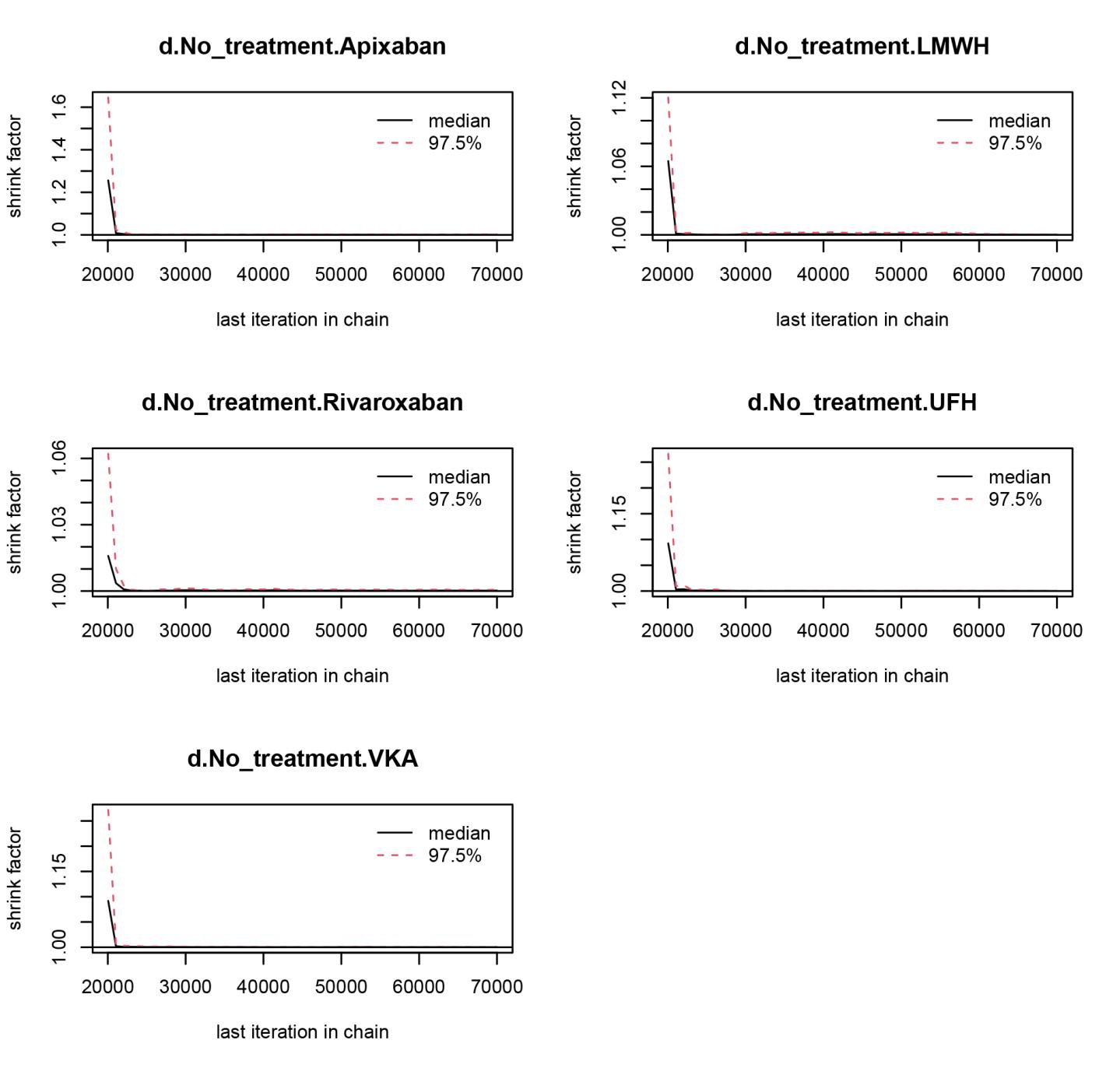
**

**Supplementary Figure 15.** Diagnostic plots: major bleeding. The shrink factor is the PSRF value, and the closer it is to 1, the better the convergence is, and the model is considered to have good stability and reliable results.

**
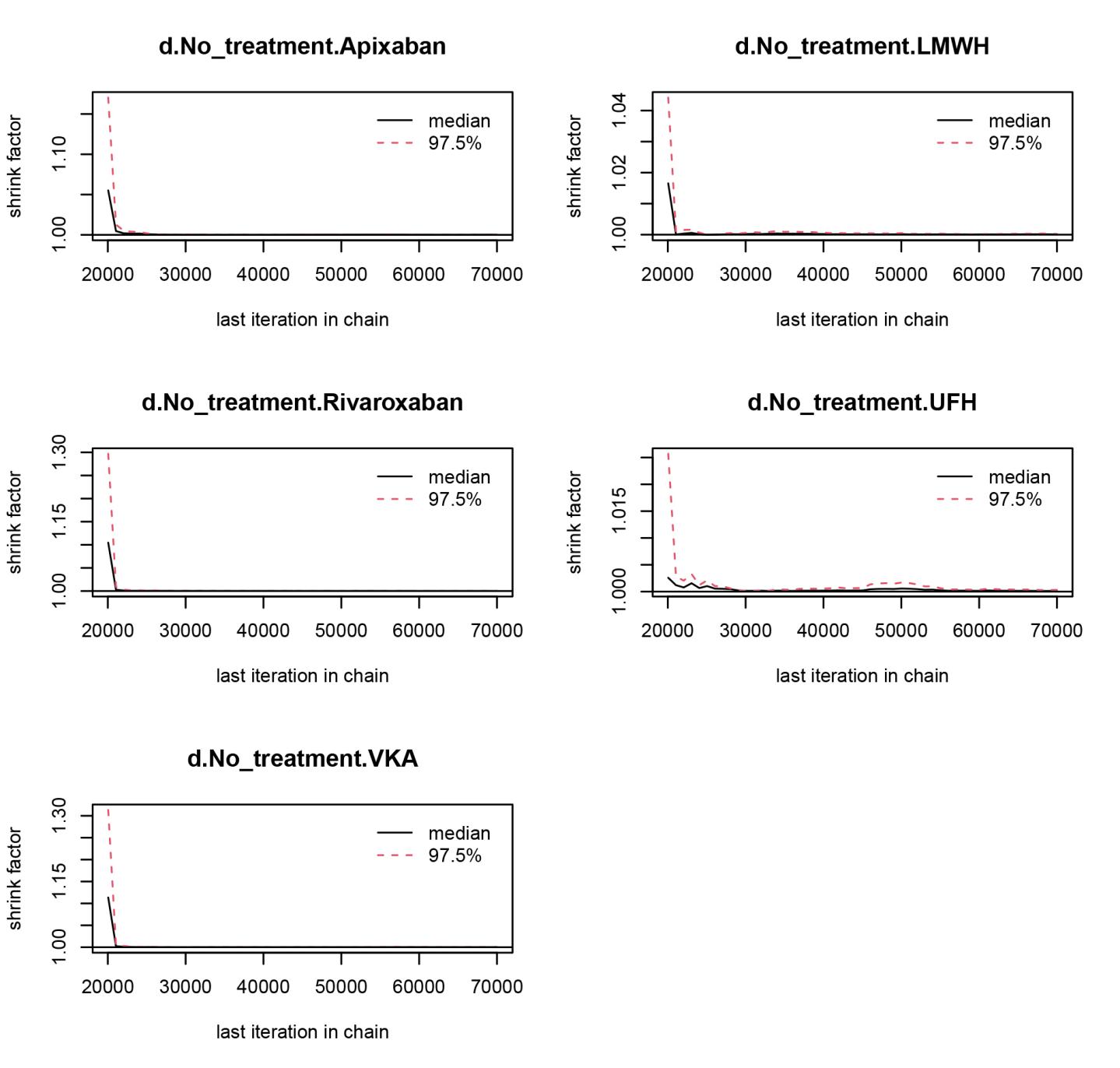
**

**Supplementary Figure 16.** Diagnostic plots: bleeding. The shrink factor is the PSRF value, and the closer it is to 1, the better the convergence is, and the model is considered to have good stability and reliable results.

**
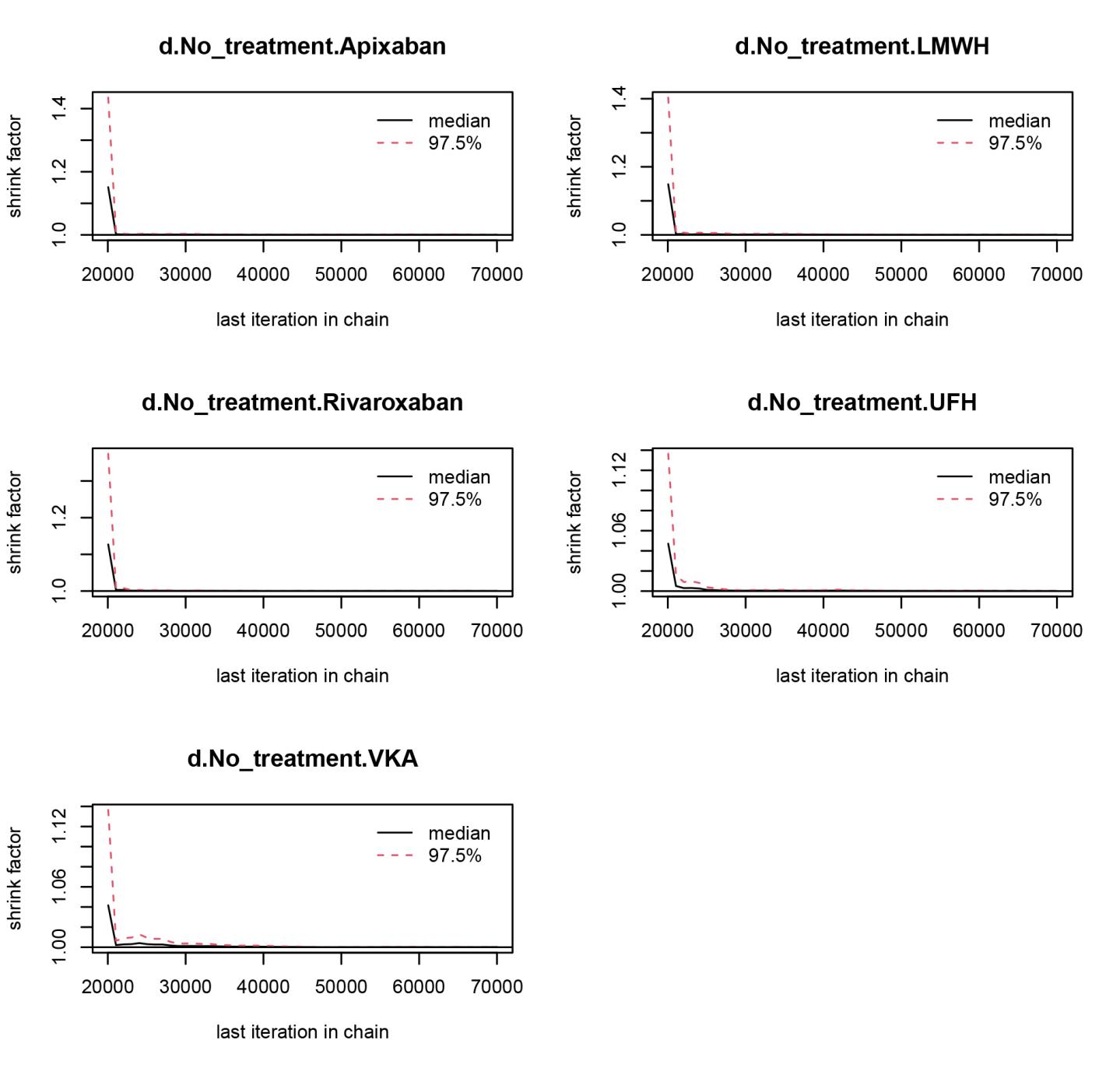
**

**Supplementary Figure 17.** Diagnostic plots: all-cause mortality. The shrink factor is the PSRF value, and the closer it is to 1, the better the convergence is, and the model is considered to have good stability and reliable results.


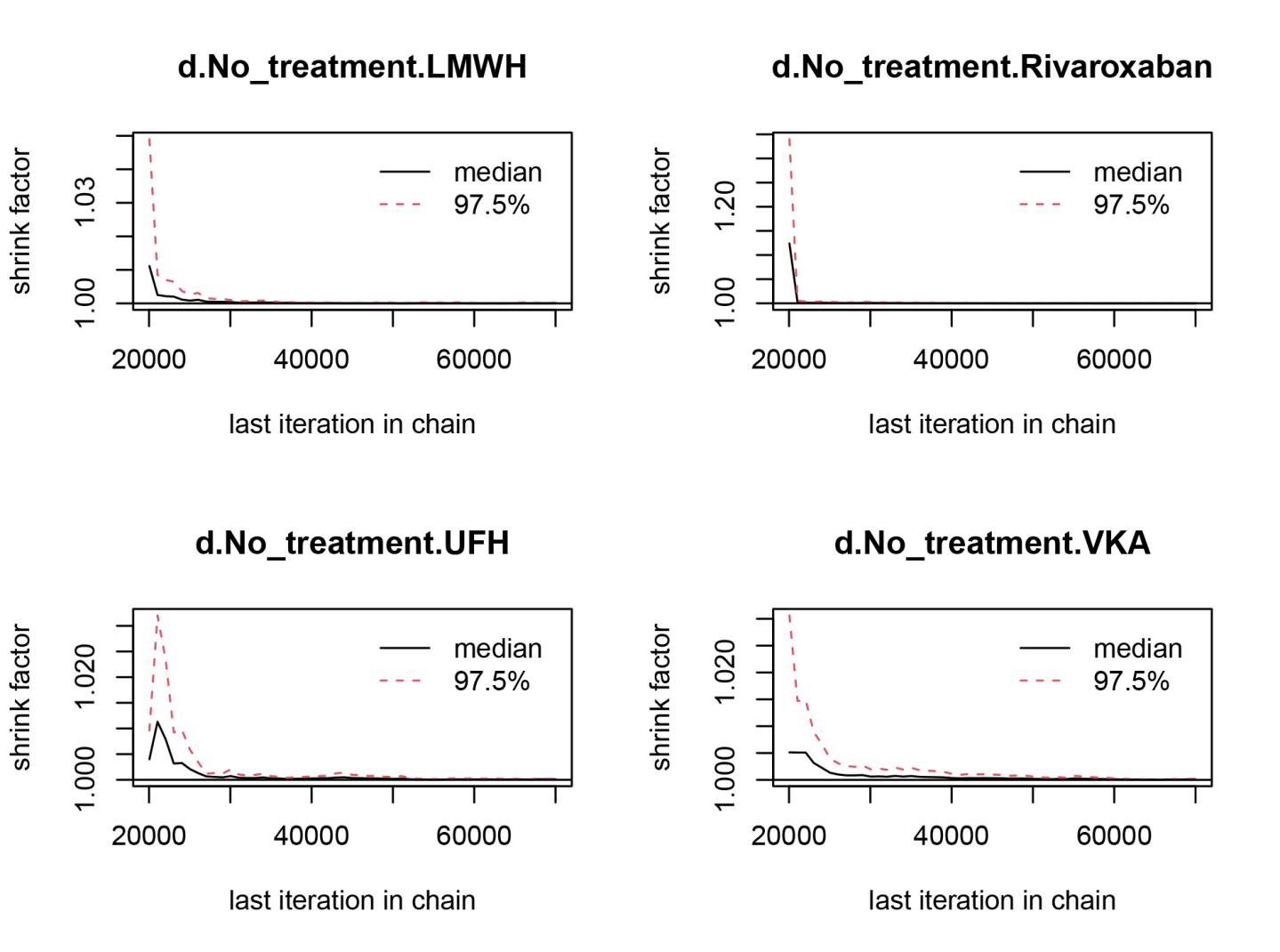


**Supplementary Figure 18.** Diagnostic plots: adverse events. The shrink factor is the PSRF value, and the closer it is to 1, the better the convergence is, and the model is considered to have good stability and reliable results.


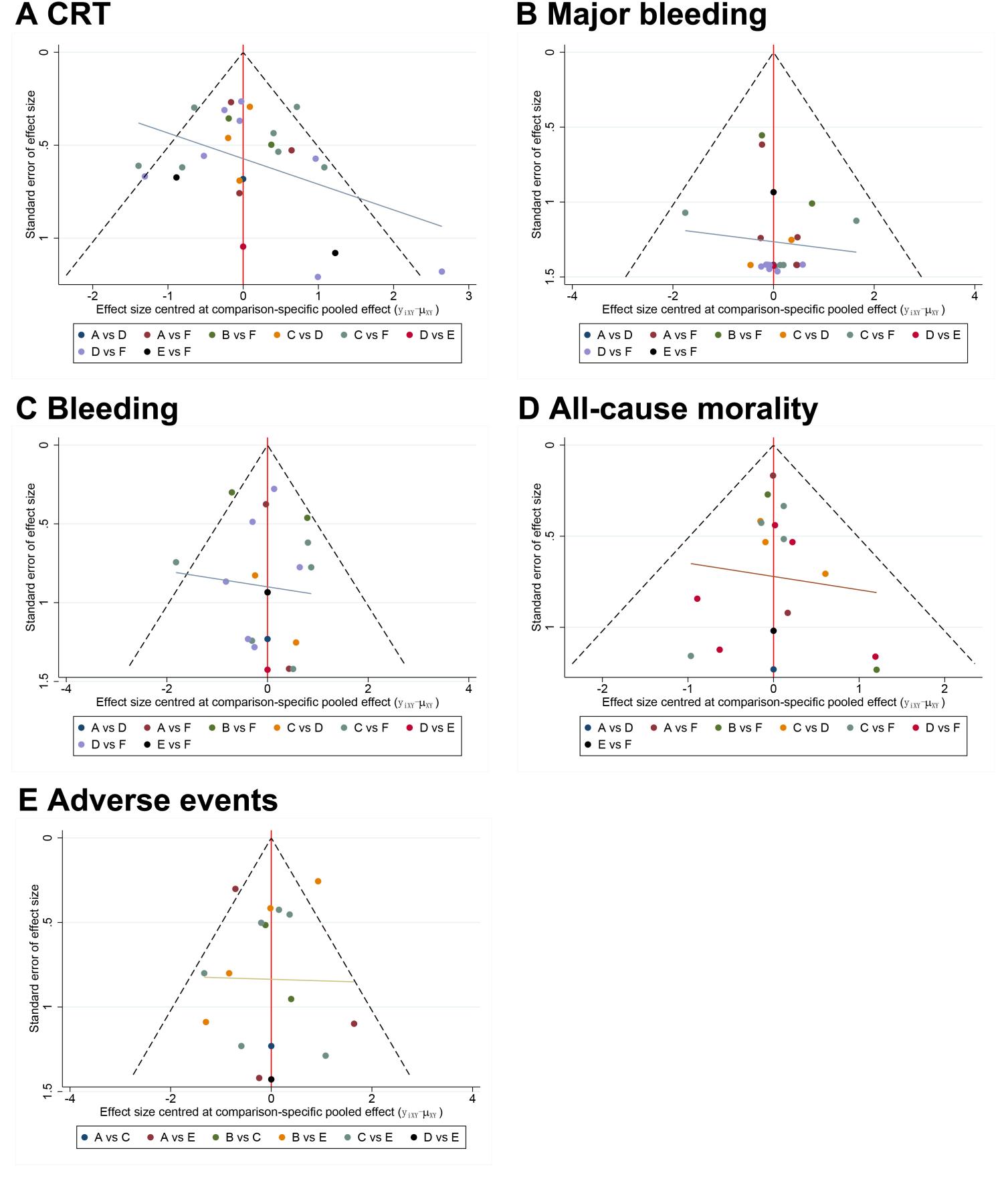


**Supplementary Figure 19.** Funnel plots.

**Supplementary Table 1.** PRISMA 2020 Checklist

| **Section and Topic** | **Item #** | **Checklist item** |
| --- | --- | --- |
| **TITLE** | | |
| Title | 1 | Identify the report as a systematic review. |
| **ABSTRACT** | | |
| Abstract | 2 | See the PRISMA 2020 for Abstracts checklist. |
| **INTRODUCTION** | | |
| Rationale | 3 | Describe the rationale for the review in the context of existing knowledge. |
| Objectives | 4 | Provide an explicit statement of the objective(s) or question(s) the review addresses. |
| **METHODS** | | |
| Eligibility criteria | 5 | Specify the inclusion and exclusion criteria for the review and how studies were grouped for the syntheses. |
| Information sources | 6 | Specify all databases, registers, websites, organisations, reference lists and other sources searched or consulted to identify studies. Specify the date when each source was last searched or consulted. |
| Search strategy | 7 | Present the full search strategies for all databases, registers and websites, including any filters and limits used. |
| Selection process | 8 | Specify the methods used to decide whether a study met the inclusion criteria of the review, including how many reviewers screened each record and each report retrieved, whether they worked independently, and if applicable, details of automation tools used in the process. |
| Data collection process | 9 | Specify the methods used to collect data from reports, including how many reviewers collected data from each report, whether they worked independently, any processes for obtaining or confirming data from study investigators, and if applicable, details of automation tools used in the process. |
| Data items | 10a | List and define all outcomes for which data were sought. Specify whether all results that were compatible with each outcome domain in each study were sought (e.g. for all measures, time points, analyses), and if not, the methods used to decide which results to collect. |
|  | 10b | List and define all other variables for which data were sought (e.g. participant and intervention characteristics, funding sources). Describe any assumptions made about any missing or unclear information. |
| Study risk of bias assessment | 11 | Specify the methods used to assess risk of bias in the included studies, including details of the tool(s) used, how many reviewers assessed each study and whether they worked independently, and if applicable, details of automation tools used in the process. |
| Effect measures | 12 | Specify for each outcome the effect measure(s) (e.g. risk ratio, mean difference) used in the synthesis or presentation of results. |
| Synthesis methods | 13a | Describe the processes used to decide which studies were eligible for each synthesis (e.g. tabulating the study intervention characteristics and comparing against the planned groups for each synthesis (item #5)). |
|  | 13b | Describe any methods required to prepare the data for presentation or synthesis, such as handling of missing summary statistics, or data conversions. |
|  | 13c | Describe any methods used to tabulate or visually display results of individual studies and syntheses. |
|  | 13d | Describe any methods used to synthesize results and provide a rationale for the choice(s). If meta-analysis was performed, describe the model(s), method(s) to identify the presence and extent of statistical heterogeneity, and software package(s) used. |
|  | 13e | Describe any methods used to explore possible causes of heterogeneity among study results (e.g. subgroup analysis, meta-regression). |
|  | 13f | Describe any sensitivity analyses conducted to assess robustness of the synthesized results. |
| Reporting bias assessment | 14 | Describe any methods used to assess risk of bias due to missing results in a synthesis (arising from reporting biases). |
| Certainty assessment | 15 | Describe any methods used to assess certainty (or confidence) in the body of evidence for an outcome. |
| **RESULTS** | | |
| Study selection | 16a | Describe the results of the search and selection process, from the number of records identified in the search to the number of studies included in the review, ideally using a flow diagram. |
|  | 16b | Cite studies that might appear to meet the inclusion criteria, but which were excluded, and explain why they were excluded. |
| Study characteristics | 17 | Cite each included study and present its characteristics. |
| Risk of bias in studies | 18 | Present assessments of risk of bias for each included study. |
| Results of individual studies | 19 | For all outcomes, present, for each study: (a) summary statistics for each group (where appropriate) and (b) an effect estimate and its precision (e.g. confidence/credible interval), ideally using structured tables or plots. |
| Results of syntheses | 20a | For each synthesis, briefly summarise the characteristics and risk of bias among contributing studies. |
|  | 20b | Present results of all statistical syntheses conducted. If meta-analysis was done, present for each the summary estimate and its precision (e.g. confidence/credible interval) and measures of statistical heterogeneity. If comparing groups, describe the direction of the effect. |
|  | 20c | Present results of all investigations of possible causes of heterogeneity among study results. |
|  | 20d | Present results of all sensitivity analyses conducted to assess the robustness of the synthesized results. |
| Reporting biases | 21 | Present assessments of risk of bias due to missing results (arising from reporting biases) for each synthesis assessed. |
| Certainty of evidence | 22 | Present assessments of certainty (or confidence) in the body of evidence for each outcome assessed. |
| **DISCUSSION** | | |
| Discussion | 23a | Provide a general interpretation of the results in the context of other evidence. |
|  | 23b | Discuss any limitations of the evidence included in the review. |
|  | 23c | Discuss any limitations of the review processes used. |
|  | 23d | Discuss implications of the results for practice, policy, and future research. |
| **OTHER INFORMATION** | | |
| Registration and protocol | 24a | Provide registration information for the review, including register name and registration number, or state that the review was not registered. |
|  | 24b | Indicate where the review protocol can be accessed, or state that a protocol was not prepared. |
|  | 24c | Describe and explain any amendments to information provided at registration or in the protocol. |
| Support | 25 | Describe sources of financial or non-financial support for the review, and the role of the funders or sponsors in the review. |
| Competing interests | 26 | Declare any competing interests of review authors. |
| Availability of data, code and other materials | 27 | Report which of the following are publicly available and where they can be found: template data collection forms; data extracted from included studies; data used for all analyses; analytic code; any other materials used in the review. |

**Supplementary Table 2.** Search strategy for bibliometric analysis

| Web of Science | | |
| --- | --- | --- |
| #1 | (((((((((((((((((((((((((((((((((((((((((((((((TS=(carcinoma)) OR TS=(sarcoma) OR TS=(Neoplasms)) OR TS=(Neoplasm)) OR TS=(Tumor)) OR TS=(Neoplasia)) OR TS=(Cancer)) OR TS=(Malignancy)) OR TS=(Malignancies)) OR TS=(Benign Neoplasm)) OR TS=(oncomap)) OR TS=(ade-nocarcinoma)) OR TS=(tumour)) OR TS=(oncology)) OR TS=(arcin*)) OR TS=(cancer*)) OR TS=(neoplas*)) OR TS=(tumour*)) OR TS=(tumor*)) OR TS=(cyst*)) OR TS=(growth*)) OR TS=(adenocarcin*)) OR TS=(malignant)) OR TS=(malig*)) OR TS=(sarcoma)) OR TS=(melanoma)) OR TS=(osteosarcoma)) OR TS=(mesothelioma)) OR TS=(glioblastoma)) OR TS=(glioblastomas)) OR TS=(glioma)) OR TS=(Gliomas)) OR TS=(melanoma)) OR TS=(osteosarcoma)) OR TS=(hepatoblastoma)) OR TS=(Lymphoma)) OR TS=(chondrosarcoma)) OR TS=(meningioma)) OR TS=(choriocarcinoma)) OR TS=(fibrosarcoma)) OR TS=(liposarcoma)) OR TS=(Hemangiosarcoma)) OR TS=(Angiosarcoma)) OR TS=(mesothelioma)) OR TS=(leiomyosarcoma)) OR TS=(rhabdomyosarcoma)) OR TS=(cystadenocarcinoma)) OR TS=(seminoma)) | [7,035,233](https://www.webofscience.com/wos/alldb/summary/1d4c4153-cba0-481e-992f-94b265136cfe-a409f4dd/relevance/1) |
| #2 | (((((((((((((((((((((((((((((((((((((((((((((((((TS=(dalteparin)) OR TS=(Tedelparin)) OR TS=(Dalteparin Sodium)) OR TS=(Fragmin)) OR TS=(Fragmine)) OR TS=(Low molecular weight heparin)) OR TS=(LMWH)) OR TS=(Heparin)) OR TS=(Unfractionated Heparin)) OR TS=(Heparinic Acid)) OR TS=(Liquaemin)) OR TS=(Sodium Heparin)) OR TS=(Heparin Sodium)) OR TS=(alpha Heparinheparin )) OR TS=(Oral Factor Xa Inhibitor)) OR TS=(Fondaparinux)) OR TS=(Fondaparinux Sodium)) OR TS=(Quixidar)) OR TS=(Arixtra)) OR TS=(Arixtra)) OR TS=(Nadroparin)) OR TS=(Nadroparine)) OR TS=(Nadroparin Calcium)) OR TS=(Fraxiparine OR Enoxaparin)) OR TS=(fraxiparin)) OR TS=(clexane)) OR TS=(lovenox )) OR TS=(fragmin)) OR TS=(ardeparin)) OR TS=(normiﬂo)) OR TS=(tinzaparin)) OR TS=(danaproid)) OR TS=(orgaran)) OR TS=(Pradaxa)) OR TS=(Dabigatran)) OR TS=(rivaroxaban)) OR TS=(Xarelto)) OR TS=(apixaban)) OR TS=(edoxaban)) OR TS=(Warfarin)) OR TS=(Eliquis)) OR TS=(coumadin)) OR TS=(acenocoumarol)) OR TS=(phenprocumon)) OR TS=(oral anticoagulant)) OR TS=(vitamin K antagonist)) OR TS=(VKA)) OR TS=(anticoagulation)) OR TS=(anticoagulant)) | 192,397 |
| #3 | ((((((((((TS=(peripherally inserted central catheters)) OR TS=(PICC)) OR TS=(peripheral central venous catheter)) OR TS=(central venous catheter)) OR TS=(CVC)) OR TS=(tunneled central venous catheter)) OR TS=(CVTC)) OR TS=(infusion port)) OR TS=(transfusion port) OR TS=(PORT)) OR TS=(totally implantable venous access device)) | 98,595 |
| #4 | #1 AND #2 AND #3 | 711 |

**Supplementary Table 3.** Search strategy for network meta-analysis

| PubMed | | | | | | | | |
| --- | --- | --- | --- | --- | --- | --- | --- | --- |
| #1 | | Neoplasm[MeSH] OR carcinoma[MeSH] OR sarcoma[MeSH]OR Neoplasms[tiab] OR Neoplasm[tiab] OR Tumor[tiab] OR Neoplasm[tiab] OR Neoplasia[tiab] OR Cancer[tiab] OR Malignant[tiab] OR Malignancy[tiab] OR Malignancies[tiab] OR Benign Neoplasm[tiab] OR oncoma[tiab] OR carcinoma[tiab] OR ade-nocarcinoma[tiab] OR tumour[tiab] OR oncology[tiab] OR arcin*[tiab] OR cancer*[tiab] OR neoplas*[tiab] OR tumour*[tiab] OR tumor*[tiab] OR cyst*[tiab] OR growth*[tiab] OR adenocarcin*[tiab] OR malignant[tiab] OR malig*[tiab] OR sarcoma[tiab] OR glioblastoma[tiab] OR glioblastomas[tiab] OR glioma[tiab] OR Gliomas[tiab] OR meningioma[tiab] OR choriocarcinoma[tiab] OR fibrosarcoma[tiab] OR liposarcoma[tiab] OR Hemangiosarcoma[tiab] OR Angiosarcoma[tiab] OR leiomyosarcoma[tiab] OR rhabdomyosarcoma[tiab] OR cystadenocarcinoma[tiab] OR seminoma[tiab] | | | | | | 7,075,696 |
| #2 | | apixaban [Supplementary Concept] OR edoxaban [Supplementary Concept] OR Warfarin[MeSH] OR dalteparin[MeSH] OR Enoxaparin[Mesh] OR dalteparin[tiab] OR Tedelparin[tiab] OR Dalteparin Sodium[tiab] OR Fragmin[tiab] OR Fragmine[tiab] OR Low molecular weight heparin[tiab] OR LMWH[tiab] OR Heparin[tiab] OR Unfractionated Heparin[tiab] OR alpha Heparinheparin [tiab] OR Oral Factor Xa Inhibitor[tiab] OR Fondaparinux[tiab] OR Fondaparinux Sodium[tiab] OR Quixidar[tiab] OR Arixtra[tiab] OR Arixtra[tiab] OR Nadroparin[tiab] OR Nadroparine[tiab] OR Nadroparin Calcium[tiab] OR Fraxiparine OR Enoxaparin[tiab] OR fraxiparin[tiab] OR clexane[tiab] OR lovenox [tiab] OR fragmin[tiab] OR ardeparin[tiab] OR normiﬂo[tiab] OR tinzaparin[tiab] OR Dabigatran[tiab] OR rivaroxaban[tiab] OR Xarelto[tiab] OR apixaban[tiab] OR edoxaban[tiab] OR melagatran[tiab] OR Coumarin[tiab] OR Warfarin[tiab] OR oral anticoagulant[tiab] OR vitamin K antagonist[tiab] OR VKA[tiab] OR Bivalirudin[tiab] OR Angiomax[tiab] | | | | | | 160,898 |
| #3 | | Catheterization, Peripheral[MeSH] OR Peripheral Catheterization[tiab] OR Catheterizations, Peripheral[tiab] OR Peripheral Catheterizations[tiab] OR Catheterization, Bronchial[tiab] OR Bronchial Catheterization[tiab] OR Bronchial Catheterizations[tiab] OR Peripheral Venous Catheterization[tiab] OR Catheterizations, Peripheral Venous[tiab] OR Peripheral Venous Catheterizations[tiab] OR PICC[tiab] OR Central Venous Catheters[MeSH] OR Central Venous Catheter[tiab] OR peripherally inserted central catheters[tiab] OR PICC[tiab] OR peripheral central venous catheter[tiab] OR central venous catheter[tiab] OR CVC[tiab] OR CVTC[tiab] OR PORT[tiab] OR totally implantable venous access device[tiab] | | | | | | 47,627 |
| #4 | | (randomized controlled trial[Publication Type] OR controlled clinical trial[Publication Type] OR randomized[tiab] OR placebo[tiab] OR Clinical Trials as Topic[Mesh:NoExp] OR randomly[tiab] OR trial[tiab] OR Prospective Studies[MeSH] OR Observational Study [Publication Type] OR Observational Studies as Topic[MeSH] OR Case-Control Studies[MeSH] OR Retrospective Studies[MeSH] OR Case-Control Studies[tiab] OR case control study[tiab] OR Cohort Studies[tiab] OR cohort study[tiab] OR cohort analy*[tiab] OR Retrospective Studies[tiab] OR retrospective[tiab] OR observational study[tiab] OR Follow up study[tiab] OR Follow up studies[tiab] OR longitudinal[tiab] OR observational studies[tiab] OR case control[tiab]) NOT (Animals[Mesh] NOT Humans[Mesh]) | | | | | | 4,715,920 |
| #5 | | #1 AND #2 AND #3 AND #4 | | | | | | 171 |
| Web of Science | | | | | | | | |
| #1 | | | | (((((((((((((((((((((((((((((((((((((((((((((((TS=(carcinoma)) OR TS=(sarcoma) OR TS=(Neoplasms)) OR TS=(Neoplasm)) OR TS=(Tumor)) OR TS=(Neoplasia)) OR TS=(Cancer)) OR TS=(Malignancy)) OR TS=(Malignancies)) OR TS=(Benign Neoplasm)) OR TS=(oncomap)) OR TS=(ade-nocarcinoma)) OR TS=(tumour)) OR TS=(oncology)) OR TS=(arcin*)) OR TS=(cancer*)) OR TS=(neoplas*)) OR TS=(tumour*)) OR TS=(tumor*)) OR TS=(cyst*)) OR TS=(growth*)) OR TS=(adenocarcin*)) OR TS=(malignant)) OR TS=(malig*)) OR TS=(sarcoma)) OR TS=(melanoma)) OR TS=(osteosarcoma)) OR TS=(mesothelioma)) OR TS=(glioblastoma)) OR TS=(glioblastomas)) OR TS=(glioma)) OR TS=(Gliomas)) OR TS=(melanoma)) OR TS=(osteosarcoma)) OR TS=(hepatoblastoma)) OR TS=(Lymphoma)) OR TS=(chondrosarcoma)) OR TS=(meningioma)) OR TS=(choriocarcinoma)) OR TS=(fibrosarcoma)) OR TS=(liposarcoma)) OR TS=(Hemangiosarcoma)) OR TS=(seminoma)) | 13,316,417 | | | |
| #2 | | | | (((((((((((((((((((((((((((((((((((((((((((((((((TS=(dalteparin)) OR TS=(Tedelparin)) OR TS=(Dalteparin Sodium)) OR TS=(Fragmin)) OR TS=(Fragmine)) OR TS=(Low molecular weight heparin)) OR TS=(LMWH)) OR TS=(Heparin)) OR TS=(Unfractionated Heparin)) OR TS=(Heparinic Acid)) OR TS=(Liquaemin)) OR TS=(Sodium Heparin)) OR TS=(Heparin Sodium)) OR TS=(alpha Heparinheparin )) OR TS=(Oral Factor Xa Inhibitor)) OR TS=(Fondaparinux)) OR TS=(Fondaparinux Sodium)) OR TS=(Quixidar)) OR TS=(Arixtra)) OR TS=(Arixtra)) OR TS=(Nadroparin)) OR TS=(Nadroparine)) OR TS=(Nadroparin Calcium)) OR TS=(Fraxiparine OR Enoxaparin)) OR TS=(fraxiparin)) OR TS=(clexane)) OR TS=(lovenox )) OR TS=(fragmin)) OR TS=(ardeparin)) OR TS=(normiﬂo)) OR TS=(tinzaparin)) OR TS=(danaproid)) OR TS=(orgaran)) OR TS=(Pradaxa)) OR TS=(Dabigatran)) OR TS=(rivaroxaban)) OR TS=(Xarelto)) OR TS=(apixaban)) OR TS=(edoxaban)) OR TS=(Warfarin)) OR TS=(Eliquis)) OR TS=(coumadin)) OR TS=(acenocoumarol)) OR TS=(phenprocumon)) OR TS=(oral anticoagulant)) OR TS=(vitamin K antagonist)) OR TS=(VKA)) OR TS=(anticoagulation)) OR TS=(anticoagulant)) | 400,991 | | | |
| #3 | | | | ((((((((((TS=(peripherally inserted central catheters)) OR TS=(PICC)) OR TS=(peripheral central venous catheter)) OR TS=(central venous catheter)) OR TS=(CVC)) OR TS=(tunneled central venous catheter)) OR TS=(CVTC)) OR TS=(infusion port)) OR TS=(transfusion port) OR TS=(PORT)) OR TS=(totally implantable venous access device)) | 166,453 | | | |
| #4 | | | | (((((((((((((((((((((((TS=(randomized controlled trial)) OR TS=(controlled clinical trial)) OR TS=(randomized)) OR TS=(placebo)) OR TS=(randomly)) OR TS=(trial)) OR TS=(Clinical Trials)) OR TS=(Prospective Studies)) OR TS=(Prospective Study)) OR TS=(prospective)) OR TS=(Case-Control Studies)) OR TS=(case control study)) OR TS=(Cohort Studies)) OR TS=(cohort study)) OR TS=( cohort analy*)) OR TS=(Retrospective Studies)) OR TS=(retrospective)) OR TS=(observational study)) OR TS=(Follow up study)) OR TS=(Follow up studies)) OR TS=(longitudinal)) OR TS=(observational studies)) OR TS=(case control)) | 9,125,379 | | | |
| #5 | | | | #1 AND #2 AND #3 AND #4 | 858 | | | |
| Embase | | | | | | | | |
| #1 | neoplasms'/exp | | | | | | 6468743 | |
| #2 | Neoplasms':ab,kw,ti OR 'Neoplasm':ab,kw,ti OR 'Tumor':ab,kw,ti OR 'Neoplasm':ab,kw,ti OR 'Neoplasia':ab,kw,ti OR 'Cancer':ab,kw,ti OR 'Malignant':ab,kw,ti OR 'Malignancy':ab,kw,ti OR 'Malignancies':ab,kw,ti OR 'Benign Neoplasm':ab,kw,ti OR 'oncoma':ab,kw,ti OR 'carcinoma':ab,kw,ti OR 'tumour':ab,kw,ti OR 'oncology':ab,kw,ti OR 'arcin*':ab,kw,ti OR 'cancer*':ab,kw,ti OR 'neoplas*':ab,kw,ti OR 'tumour*':ab,kw,ti OR 'tumor*':ab,kw,ti OR 'cyst*':ab,kw,ti OR 'growth*':ab,kw,ti OR 'adenocarcin*':ab,kw,ti OR 'malignant':ab,kw,ti OR 'malig*':ab,kw,ti OR 'sarcoma':ab,kw,ti OR 'glioma':ab,kw,ti OR 'Gliomas':ab,kw,ti OR 'melanoma':ab,kw,ti OR 'Hemangiosarcoma':ab,kw,ti OR 'Angiosarcoma':ab,kw,ti OR 'mesothelioma':ab,kw,ti OR 'leiomyosarcoma':ab,kw,ti OR 'rhabdoyosarcoma':ab,kw,ti OR 'cystadenocarcinoma':ab,kw,ti OR 'seminoma':ab,kw,ti | | | | | | 8,223,421 | |
| #3 | #1 OR #2 | | | | | | 9,001,709 | |
| #4 | apixaban'/exp | | | | | | 24,043 | |
| #5 | rivaroxaban'/exp | | | | | | 31,113 | |
| #6 | dalteparin':ab,kw,ti OR 'Tedelparin':ab,kw,ti OR 'Fragmin':ab,kw,ti OR 'Fragmine':ab,kw,ti OR 'Low molecular weight heparin':ab,kw,ti OR 'LMWH':ab,kw,ti OR 'Heparin':ab,kw,ti OR 'Unfractionated Heparin':ab,kw,ti OR 'Heparinic Acid':ab,kw,ti OR 'Liquaemin':ab,kw,ti OR 'Sodium Heparin':ab,kw,ti OR 'Heparin Sodium':ab,kw,ti OR 'alpha Heparinheparin':ab,kw,ti OR 'Fondaparinux':ab,kw,ti OR 'Fondaparinux Sodium':ab,kw,ti OR 'Nadroparin':ab,kw,ti OR 'Nadroparine':ab,kw,ti OR 'Nadroparin Calcium':ab,kw,ti OR 'Fraxiparine':ab,kw,ti OR 'Enoxaparin':ab,kw,ti OR 'Pradaxa':ab,kw,ti OR 'Dabigatran':ab,kw,ti OR 'rivaroxaban':ab,kw,ti OR 'Xarelto':ab,kw,ti OR 'apixaban':ab,kw,ti OR 'edoxaban':ab,kw,ti OR 'ximelagatran':ab,kw,ti OR 'Warfarin':ab,kw,ti OR 'oral anticoagulant':ab,kw,ti OR 'vitamin K antagonist':ab,kw,ti OR 'VKA':ab,kw,ti OR 'Bivalirudin':ab,kw,ti OR 'Angiomax':ab,kw,ti | | | | | | 210,807 | |
| #7 | #4 OR #5 OR #6 | | | | | | 9,227,132 | |
| #8 | totally implantable venous access device'/exp | | | | | | 50 | |
| #9 | central venous catheter'/exp | | | | | | 36,117 | |
| #10 | peripherally inserted central catheters':ab,kw,ti OR 'PICC':ab,kw,ti OR 'peripheral central venous catheter':ab,kw,ti OR 'central venous catheter':ab,kw,ti OR 'CVC':ab,kw,ti OR 'tunneled central venous catheter':ab,kw,ti OR 'infusion port':ab,kw,ti OR 'transfusion port':ab,kw,ti OR 'PORT':ab,kw,ti OR 'totally implantable venous access device':ab,kw,ti | | | | | | 76,282 | |
| #11 | #8 OR #9 OR #10 | | | | | | 3,135,517 | |
| #12 | randomized controlled trial'/exp | | | | | | 854,750 | |
| #13 | randomized controlled trial':ab,kw,ti OR 'controlled clinical trial':ab,kw,ti OR 'randomized':ab,kw,ti OR 'placebo':ab,kw,ti OR 'randomly':ab,kw,ti OR 'trial':ab,kw,ti OR 'Clinical Trials':ab,kw,ti OR 'Prospective Studies':ab,kw,ti OR 'Prospective Study':ab,kw,ti OR 'prospective':ab,kw,ti OR 'Case-Control Studies':ab,kw,ti OR 'case control study':ab,kw,ti OR 'Cohort Studies':ab,kw,ti OR 'cohort study':ab,kw,ti OR ' cohort analy*':ab,kw,ti OR 'Retrospective Studies':ab,kw,ti OR 'retrospective':ab,kw,ti OR 'observational study':ab,kw,ti OR 'Follow up study':ab,kw,ti OR 'Follow up studies':ab,kw,ti OR 'longitudinal':ab,kw,ti OR 'observational studies':ab,kw,ti OR 'case control':ab,kw,ti | | | | | | 5,608,260 | |
| #14 | #12 OR #13 | | | | | | 5,715,303 | |
| #15 | #3 AND #7 AND #11 AND #14 | | | | | | 499 | |
| Cochranne | | | | | | | | |
| #1 | | | MeSH descriptor: [Neoplasms] explode all trees | | | 127,536 | | |
| #2 | | | ("Neoplasms" OR "Neoplasm" OR "Tumor" OR "Neoplasm" OR "Neoplasia" OR "Cancer" OR "Malignant" OR "Malignancy" OR "Malignancies" OR "Benign Neoplasm" OR "oncoma" OR "carcinoma" OR "ade-nocarcinoma" OR "tumour" OR "oncology" OR "malignant" OR "sarcoma" OR "glioblastoma" OR "glioblastomas" OR "glioma" OR "Gliomas" OR "melanoma" OR "osteosarcoma" OR "hepatoblastoma" OR "Lymphoma" OR "chondrosarcoma" OR "meningioma" OR "choriocarcinoma" OR "fibrosarcoma" OR "Hemangiosarcoma" OR "Angiosarcoma" OR "mesothelioma" OR "leiomyosarcoma" OR "rhabdoyosarcoma" OR "cystadenocarcinoma" OR "seminoma"):ab,kw,ti | | | 294,525 | | |
| #3 | | | #1 OR #2 | | | 303,673 | | |
| #4 | | | MeSH descriptor: [Rivaroxaban] explode all trees | | | 1,005 | | |
| #5 | | | MeSH descriptor: [Heparin, Low-Molecular-Weight] explode all trees | | | 2,760 | | |
| #6 | | | MeSH descriptor: [Heparin] explode all trees | | | 6,287 | | |
| #7 | | | (“dalteparin" OR "Tedelparin" OR "Dalteparin Sodium" OR "Fragmin" OR "Fragmine" OR "Low molecular weight heparin" OR "LMWH" OR "Heparin" OR "Unfractionated Heparin" OR "Heparinic Acid" OR "Liquaemin" OR "Sodium Heparin" OR "Heparin Sodium" OR "alpha Heparinheparin " OR "Oral Factor Xa Inhibitor" OR "Fondaparinux" OR "Fondaparinux Sodium" OR "Quixidar" OR "Arixtra" OR "Arixtra" OR "Nadroparin" OR " Nadroparine" OR " Nadroparin Calcium" OR " Fraxiparine OR Enoxaparin" OR "fraxiparin" OR "clexane" OR "lovenox " OR "fragmin" OR "ardeparin" OR " normiﬂo" OR "tinzaparin" OR "logiparin" OR "innohep" OR "certoparin" OR "sandoparin" OR "reviparin" OR "clivarin" OR "danaproid" OR "orgaran" OR "Pradaxa" OR "Dabigatran" OR "rivaroxaban" OR " apixaban" OR "edoxaban" OR "blood clotting factor 10a inhibitor" OR "betrixaban" OR "Coumarins" OR "Warfarin" OR "Eliquis" OR "coumadin" OR "acenocoumarol" OR "phenprocumon" OR "oral anticoagulant" OR "vitamin K antagonist" OR "VKA" OR "Bivalirudin" OR "Angiomax”):ab,kw,ti | | | 21,664 | | |
| #8 | | | #4 OR #5 OR #6 OR #7 | | | 22,918 | | |
| #9 | | | MeSH descriptor: [Central Venous Catheters] explode all trees | | | 322 | | |
| #10 | | | (“peripherally inserted central catheters" OR "PICC" OR "peripheral central venous catheter" OR "central venous catheter" OR "CVC" OR "tunneled central venous catheter" OR "CVTC" OR "infusion port" OR "transfusion port" OR "PORT" OR "totally implantable venous access device”):ab,kw,ti | | | 6,388 | | |
| #11 | | | #9 OR #10 | | | 6,461 | | |
| #12 | | | ("Randomized Controlled Trial " OR "RCT" OR " Controlled Clinical Trial " OR " Random Allocation " OR " Placebos " OR " Random " OR " Randomization " OR " Trial"):ti,ab,kw | | | 1,220,170 | | |
| #13 | | | ("Animals" NOT "Humans"):ti,ab,kw | | | 5,231 | | |
| #14 | | | #12 NOT #13 | | | 1,216,257 | | |
| #15 | | | #3 AND #8 AND #11 AND #14 | | | 98 | | |

**Supplementary Table 4.** Characteristics of included studies

| **Study** | **Study design** | **Country** | **Time period** | **Sample size**  **(M/F)** | **Age** | **Intervention** | | **Duration of treatment** | **Dosages** | **Cancer type**  **(%)** | **Follow up** | **Outcomes** |
| --- | --- | --- | --- | --- | --- | --- | --- | --- | --- | --- | --- | --- |
|  |  |  |  |  |  | **T** | **C** |  |  |  |  |  |
| Bern 1990 | RCT | USA | NR | 82  (49/42) | T: 56 ± 13.5 C: 60.6 ± 10.7 | Warfarin | No treatment | 90 days | 1 mg daily | Gastrointestinal (34%), Breast (17%), Lung (13%), Other (36%) | 90 days | a |
| Monreal 1996 | RCT | Spain | 1993-1995 | 29 (15/14) | T: 56 ± 14  C: 54 ± 15 | Fragmin | No treatment | 90 days | 2,500 IU daily | Gastrointestinal (59%), Breast (21%), Other (20%) | 90 days | a, b, c, e |
| Boraks 1998 | Cohort | UK | 1994-1997 | 223 | T: 48 C: 46 | Warfarin | No treatment | 90 days | 1 mg daily | Haematological (100%) | 90 days | a, b, c, e |
| Heaton 2002 | RCT | New Zealand | NR | 88  (52/36) | T: 45 C: 41 | Warfarin | No treatment | 90 days | 1 mg daily | Haematological (72%), Other (18%) | 90 days | a, b, c, e |
| Cortelezzi 2003 | Cohort | Italy | 1999-2001 | 126  (63/63) | 54 | T1: UFH T2: Nadroparin | No treatment | NR | UFH 2,500 IU or nadroparin 3,800 IU daily | Haematological (89%), Other diseases(11%) | NR | a, b, c |
| Mismetti 2003 | RCT | France | 1998-2000 | 59  (34/25) | T: 60.3 ± 9.5 C: 57.1 ± 9.0 | Nadroparin | Warfarin | 90 days | Warfarin 1 mg or nadroparin 2,850 IU daily | Gastrointestinal (51%), Breast (24%), Lung (5%), Other (20%) | 90 days | a, b, c, d, e |
| Abdelkefi 2004 | RCT | Tunisia | 2002-2003 | 108  (65/43) | T: 27 C: 28 | UFH | Normal saline solution | 直至出院 | UFH 100 IU/kg/daily or 50 ml/daily of normal saline solution | Haematological (69%),  Other disease(31%) | 直至  出院 | a, b, c, d, e |
| Couban 2005 | RCT | Canada | 1999-2002 | 255  (152/103) | T: 51 C: 52 | Warfarin | Placebo | 置入CVC到取出CVC | 1 mg daily | Haematological (68%), Gastrointestinal (10%), Other (22%) | 置入CVC到取出CVC | a, b, c, d, e |
| Verso 2005 | RCT | Italy | 2000-2003 | 285  (176/109) | T: 59.5 C: 59.1 | Enoxaparin | Placebo | 42 days | 40 mg daily | Gastrointestinal (54%), Breast (17%), Haematological (9%), Genitourinary (6%), Other (14%) | 90 days | a, b, c, d, e |
| Karthaus 2006 | RCT | Germany, Austria,  et al | 1999-2001 | 438  (181/258) | T: 55.2 ± 12.91 C: 57.4 ± 12.72 | Dalteparin | Placebo | 16 weeks | 5,000 IU daily | Solid (90%), Hematological (10%) | 16 weeks | a, b, c, d, e |
| Niers 2007 | RCT | Netherlands | NR | 113  (62/51) | T: 58 ± 10 C: 55 ± 13 | Nadroparin | Placebo | 21 days | 2,850 antifactor Xa units daily | Haematological (100%) | 21 days | a, b, c, e |
| Young 2009 | RCT | UK | 1999-2004 | 812  (499/313) | T: 60 C: 61 | Warfarin | Placebo | NR | Warfarin 1 mg daily, or dose-adjusted warfarin to maintain INR 1.5-2.0 | Gastrointestinal (76%), Breast (8%), Other (16%) | NR | a, b, c, d |
| Cicco 2009 | RCT | Italy | 2000-2004 | 450  (165/285) | T1: 55.5 T2: 55.3 C: 55.1 | T1: Acenocumarine T2: dalteparin | No treatment | 30 days | Acenocumarine 1 mg/day or dalteparin 5,000 IU | Breast (32%), Genitourinary (12%), Other (28%) | 30 days | a, b, c, d |
| Lavau-  Denes 2013 | RCT | France | 1999-2009 | 407 (243/164) | T1: 59 T2: 61 C: 60 | T1: Warfarin T2: LMWH | No treatment | 90 days | Acenocumarine 1 mg/day or LMWH, once daily | Gastrointestinal (30%), Head and neck (24%), Lung (11%), Breast (11%), Other (24%) | 90 days | a, b, c, d, e |
| Carrier 2018 | RCT | Canada | 2014-2018 | 574  (240/334) | T: 61.2 ± 12.4 C: 61.7 ± 11.3 | Apixaban | Placebo | 180 days | 2.5 mg twice daily | Haematological (28%), Gynecologic (26%), Pancreatic (14%), Lung (10%), Other (22%) | 180 days | a, b, c, d |
| Lv 2019 | Cohort | China | 2014-2015 | 423  (226/197) | T1: 57.2 ± 9.7 T2: 56.7 ± 7.7 C: 58.2 ± 8.1 | T1: Rivaroxaban  T2: Enoxaparine | No treatment | 整个化疗期 | Rivaroxaban 10mg/day or enoxaparine, 4000 anti-Xa IU/day | Gastrointestinal (36%), Lung (17%), Esophageal (17%), Other (30%) | 整个化疗期 | a, b, c, d, e |
| Khorana 2019 | RCT | USA | NR | 841  (428/413) | T: 63 C: 62 | Rivaroxaban | Placebo | 180(56) days | Rivaroxaban 10 mg daily | Pancreatic (33%), Gastrointestinal (21%), Lung (16%), Other (30%) | 56 days | a, b, c, d, e |
| Ikesaka 2021 | RCT | Canada | 2019-2020 | 105  (33/72) | T: 60.0 C: 61.6 | Rivaroxaban | No treatment | 90 days | Rivaroxaban 10 mg daily | Gastrointestinal (40%), Breast (40%), Gynecological (10%)， | 90 days | a, b, c, e |
| Brandt 2022 | RCT | Canada | NR | 217  (79/138) | T: 59.4 ± 11.7 C: 58.3 ± 11.4 | Apixaban | Placebo | 180 days | 2.5 mg twice daily | Gynecological (23%), Pancreas(21), Lymphoma (18%), Other (38%) | 180 days | a, b, c, d |

Note：a: CRT; b: Major bleeding; c: Bleeding; d: All-cause mortality; e: adverse event; NT: No treatment; T: Treatment group; C: Control Group

**Supplementary Table 5.** Top 10 countries in terms of publications.

| Rank | Countries | Publications | Citations | SCP | MCP | MCP % | Total link strength |
| --- | --- | --- | --- | --- | --- | --- | --- |
| 1 | United States | 189 | 10302 | 170 | 19 | 10.1 | 97 |
| 2 | Italy | 77 | 4781 | 69 | 8 | 10.4 | 18 |
| 3 | China | 55 | 3984 | 53 | 2 | 3.6 | 20 |
| 4 | France | 50 | 2629 | 45 | 5 | 10 | 59 |
| 5 | Canada | 44 | 540 | 36 | 8 | 18.2 | 23 |
| 6 | The Netherlands | 36 | 3023 | 28 | 8 | 22.2 | 57 |
| 7 | Germany | 32 | 2116 | 28 | 4 | 12.5 | 57 |
| 8 | United Kingdom | 23 | 2656 | 16 | 7 | 30.4 | 12 |
| 9 | Spain | 18 | 1502 | 16 | 2 | 11.1 | 56 |
| 10 | Japan | 17 | 1131 | 17 | 0 | 0 | 15 |

**Note:** SCP: Indicates that all authors of this paper are from the same country，MCP: Indicates that the authors of this paper are from multiple countries, reflecting international collaboration, MCP %: The proportion of co-authored articles published by the country. A higher value indicates that the country has more collaborative articles with other countries, suggesting a tendency for international cooperation.

**Supplementary Table 6.** Top 10 institutions in terms of the number of publications.

| Rank | Institutions | Publications | Country |
| --- | --- | --- | --- |
| 1 | Mcmaster Univ | 36 | Canada |
| 2 | APHP | 29 | France |
| 3 | Univ Paris Cite | 28 | France |
| 4 | Univ of Texas | 26 | United States |
| 5 | Utmd Anderson Cancer Center | 19 | United States |
| 6 | Univ of Ottawa | 17 | Canada |
| 7 | Univ of Amsterdam | 16 | The Netherlands |
| 8 | Harvard Univ | 15 | United States |
| 9 | Ottawa Hospital | 15 | Canada |
| 10 | Academic Medical Center Amsterdam | 14 | The Netherlands |

**Note:** APHP: Assistance Publique – Hôpitaux de Pariss.

**Supplementary Table 7.** Top 10 authors in terms of the number of publications.

| Rank | Author | Publications | Citations | H-index | Countries | Total link strength |
| --- | --- | --- | --- | --- | --- | --- |
| 1 | Philippe Debourdeau | 16 | 812 | 10 | France | 97 |
| 2 | Giancarlo Agnelli | 11 | 1170 | 8 | Italy | 18 |
| 3 | Marc Carrier | 11 | 255 | 7 | Canada | 20 |
| 4 | [Elie A Akl](https://pubmed.ncbi.nlm.nih.gov/?term=Akl+EA&cauthor_id=39117011). | 10 | 250 | 8 | United States | 59 |
| 5 | [Cheryl van de Wetering](https://pubmed.ncbi.nlm.nih.gov/?term=van+de+Wetering+C&cauthor_id=39576873) | 10 | 185 | 8 | The Netherlands | 23 |
| 6 | Marta Barba | 9 | 238 | 7 | Italy | 57 |
| 7 | [Francesca Sperati](https://pubmed.ncbi.nlm.nih.gov/?term=Sperati+F&cauthor_id=38584311) | 9 | 238 | 7 | Italy | 57 |
| 8 | [Michael B Streiff](https://pubmed.ncbi.nlm.nih.gov/?term=Streiff+MB&cauthor_id=39236759) | 8 | 502 | 8 | United States | 12 |
| 9 | [Holger J Schünemann](https://pubmed.ncbi.nlm.nih.gov/?term=Sch%C3%BCnemann+HJ&cauthor_id=38685482) | 8 | 156 | 7 | Canada | 56 |
| 10 | Melina Verso | 8 | 1002 | 7 | Italy | 15 |

**Note:** H-index: Used for the comprehensive evaluation of the quality and quantity of researcher's published papers.

| Rank | Journal | Publications | Citations | IF/JCR Quartile (2025) |
| --- | --- | --- | --- | --- |
| 1 | Thrombosis research | 30 | 864 | 3.4/Q3 |
| 2 | Journal of thrombosis and haemostasis | 23 | 1359 | 5.0/Q1 |
| 3 | Cochrane database of systematic reviews | 18 | 2536 | 9.4/Q1 |
| 4 | Journal of clinical oncology | 18 | 336 | 43.4/Q1 |
| 5 | Journal of vascular access | 14 | 312 | 1.7/Q4 |
| 6 | Journal of thrombosis and thrombolysis | 13 | 423 | 2.2/Q2 |
| 7 | Supportive care in cancer | 13 | 307 | 3.0/Q3 |
| 8 | Annals of oncology | 12 | 1072 | 65.4/Q1 |
| 9 | Seminars in thrombosis and hemostasis | 10 | 287 | 4.1/Q2 |
| 10 | Antimicrobial agents and chemotherapy | 8 | 332 | 4.5/Q1 |

**Supplementary Table 8.** Top 10 journal in terms of the number of publications.

**Supplementary Table 9.** The top 10 highly cited references

| Rank | Title of the literature | Year and journal | First author | Citations | Citations per year |
| --- | --- | --- | --- | --- | --- |
| 1 | Prevention of thalidomide- and lenalidomide-associated thrombosis in myeloma | 2008, Leukemia | Palumbo A | 651 | 36.17 |
| 2 | Venous thromboembolism associated with long-term use of central venous catheters in cancer patients | 2003, J Clin Oncol | [Verso](https://pubmed.ncbi.nlm.nih.gov/?term=Verso+M&cauthor_id=14512399) M | 419 | 18.22 |
| 3 | The epidemiology of venous thromboembolism in the community | 2008, Arterioscler Thromb Vasc Biol | John A. Heit | 385 | 15.40 |
| 4 | High incidence of thromboembolic events in patients treated with cisplatin-based chemotherapy: a large retrospective analysis | 2011, J Clin Oncol | Moore RA | 306 | 20.40 |
| 5 | Factors at admission associated with bleeding risk in medical patients: findings from the IMPROVE investigators | 2011, Chest | Decousus H | 300 | 20.00 |
| 6 | Management of occlusion and thrombosis associated with long-term indwelling central venous catheters | 2009, Lancet | Baskin JL | 273 | 16.06 |
| 7 | Upper-extremity deep vein thrombosis: a prospective registry of 592 patients | 2004, Circulation | Joffe HV | 268 | 12.18 |
| 8 | International clinical practice guidelines including guidance for direct oral anticoagulants in the treatment and prophylaxis of venous thromboembolism in patients with cancer | 2016, Lancet Oncol | Farge D | 256 | 25.60 |
| 9 | The epidemiology of venous thromboembolism in the community: implications for prevention and management | 2006, J Thromb Thrombolys | Heit Ja | 241 | 12.05 |
| 10 | Randomized placebo-controlled study of low-dose warfarin for the prevention of central venous catheter-associated thrombosis in patients with cancer | 2005, J Clin Oncol | Couban S | 239 | 11.38 |

**Supplementary Table 10.** League table of VTE: RCT-Only sensitivity analysis

| **Rivaroxaban** | **0.47 (0.23, 0.93)** | 0.73 (0.45, 1.20) | 1.10 (0.68, 1.80) | **1.53 (1.04, 2.26)** |
| --- | --- | --- | --- | --- |
| **2.12 (1.07, 4.31)** | **Apixaban** | 1.55 (0.82, 3.04) | **2.34 (1.25, 4.54)** | **3.25 (1.86, 5.89)** |
| 1.37 (0.83, 2.24) | 0.64 (0.33, 1.22) | **VKA** | **1.51 (1.05, 2.16)** | **2.09 (1.54, 2.86)** |
| 0.91 (0.56, 1.47) | **0.43 (0.22, 0.80)** | **0.66 (0.46, 0.95)** | **LMWH** | **1.39 (1.03, 1.86)** |
| **0.65 (0.44, 0.96)** | **0.31 (0.17, 0.54)** | **0.48 (0.35, 0.65)** | **0.72 (0.54, 0.97)** | **No treatment** |

**Supplementary Table 11.** League table of major bleeding: RCT-Only sensitivity analysis

| **Rivaroxaban** | 0.76 (0.17, 3.33) | 0.60 (0.13, 2.49) | 0.54 (0.10, 2.68) | 0.48 (0.14, 1.38) |
| --- | --- | --- | --- | --- |
| 1.31 (0.30, 5.95) | **Apixaban** | 0.78 (0.20, 2.97) | 0.71 (0.15, 3.21) | 0.63 (0.23, 1.59) |
| 1.68 (0.40, 7.57) | 1.28 (0.34, 5.00) | **VKA** | 0.90 (0.25, 3.27) | 0.80 (0.31, 2.01) |
| 1.86 (0.37, 9.79) | 1.41 (0.31, 6.61) | 1.11 (0.31, 4.02) | **LMWH** | 0.88 (0.27, 2.84) |
| 2.10 (0.72, 6.96) | 1.60 (0.63, 4.43) | 1.26 (0.50, 3.22) | 1.14 (0.35, 3.70) | **No treatment** |

**Supplementary Table 12.** League table of bleeding: RCT-Only sensitivity analysis

| **Rivaroxaban** | 0.60 (0.25, 1.40) | 1.42 (0.56, 3.57) | 0.91 (0.40, 2.04) | 0.62 (0.30, 1.22) |
| --- | --- | --- | --- | --- |
| 1.66 (0.71, 3.93) | **Apixaban** | **2.35 (1.08, 5.17)** | 1.51 (0.79, 2.87) | 1.03 (0.63, 1.66) |
| 0.70 (0.28, 1.79) | **0.43 (0.19, 0.92)** | **VKA** | 0.64 (0.32, 1.27) | **0.44 (0.23, 0.79)** |
| 1.10 (0.49, 2.51) | 0.66 (0.35, 1.27) | 1.56 (0.79, 3.15) | **LMWH** | 0.68 (0.44, 1.04) |
| 1.62 (0.82, 3.29) | 0.98 (0.60, 1.59) | **2.29 (1.27, 4.28)** | 1.47 (0.96, 2.27) | **No treatment** |

**Supplementary Table 13.** League table of all-cause mortality: RCT-Only sensitivity analysis

| **Rivaroxaban** | 1.49 (0.81, 2.77) | 1.46 (0.84, 2.54) | 1.49 (0.80, 2.76) | 1.25 (0.90, 1.73) |
| --- | --- | --- | --- | --- |
| 0.67 (0.36, 1.24) | **Apixaban** | 0.98 (0.50, 1.93) | 1.00 (0.48, 2.08) | 0.84 (0.50, 1.40) |
| 0.69 (0.39, 1.19) | 1.02 (0.52, 2.02) | **VKA** | 1.02 (0.60, 1.71) | 0.86 (0.55, 1.33) |
| 0.67 (0.36, 1.24) | 1.00 (0.48, 2.08) | 0.98 (0.58, 1.65) | **LMWH** | 0.84 (0.50, 1.41) |
| 0.80 (0.58, 1.11) | 1.20 (0.71, 2.02) | 1.17 (0.75, 1.83) | 1.19 (0.71, 2.02) | **No treatment** |

**Supplementary Table 14.** League table of adverse events: RCT-Only sensitivity analysis

| **Rivaroxaban** | 0.80 (0.40, 1.55) | 0.83 (0.41, 1.69) | 0.79 (0.45, 1.35) |
| --- | --- | --- | --- |
| 1.25 (0.65, 2.48) | **VKA** | 1.05 (0.60, 1.82) | 0.99 (0.67, 1.46) |
| 1.20 (0.59, 2.44) | 0.96 (0.55, 1.66) | **LMWH** | 0.94 (0.60, 1.47) |
| 1.27 (0.74, 2.22) | 1.01 (0.69, 1.49) | 1.06 (0.68, 1.66) | **No treatment** |
